# A multi-omics approach unravels metagenomic and metabolic alterations of a probiotic and synbiotic additive in rainbow trout (*Oncorhynchus mykiss*)

**DOI:** 10.1186/s40168-021-01221-8

**Published:** 2022-01-30

**Authors:** Jacob Agerbo Rasmussen, Kasper Rømer Villumsen, Madeleine Ernst, Martin Hansen, Torunn Forberg, Shyam Gopalakrishnan, M. Thomas P. Gilbert, Anders Miki Bojesen, Karsten Kristiansen, Morten Tønsberg Limborg

**Affiliations:** 1 Laboratory of Genomics and Molecular Medicine, Department of Biology, University of Copenhagen, Copenhagen, Denmark; 2 Center for Evolutionary Hologenomics, GLOBE Institute, Faculty of Health and Medical Sciences, Copenhagen, Denmark; 3 Department of Veterinary and Animal Sciences, University of Copenhagen, Veterinary Clinical Microbiology, Copenhagen, Denmark; 4 Section for Clinical Mass Spectrometry, Danish Center for Neonatal Screening, Department of Congenital Disorders, Statens Serum Institut, 2300 Copenhagen, Denmark; 5 Department of Environmental Science, Aarhus University, Aarhus, Denmark; 6 BioMar Group, Trondheim, Norway; 7 University Museum NTNU, Trondheim, Norway; 8 Institute of Metagenomics, Qingdao-Europe Advanced Institute for Life Sciences, Qingdao, China

## Abstract

**Background:**

Animal protein production is increasingly looking towards microbiome-associated services such as the design of new and better probiotic solutions to further improve gut health and production sustainability. Here, we investigate the functional effects of bacteria-based pro- and synbiotic feed additives on microbiome-associated functions in relation to growth performance in the commercially important rainbow trout (*Oncorhynchus mykiss*). We combine complementary insights from multiple omics datasets from gut content samples, including 16S bacterial profiling, whole metagenomes, and untargeted metabolomics, to investigate bacterial metagenome-assembled genomes (MAGs) and their molecular interactions with host metabolism.

**Results:**

Our findings reveal that (I) feed additives changed the microbiome and that rainbow trout reared with feed additives had a significantly reduced relative abundance of the salmonid related *Candidatus* Mycoplasma salmoninae in both the mid and distal gut content, (II) genome resolved metagenomics revealed that alterations of microbial arginine biosynthesis and terpenoid backbone synthesis pathways were directly associated with the presence of *Candidatus* Mycoplasma salmoninae, and (III) differences in the composition of intestinal microbiota among feed types were directly associated with significant changes of the metabolomic landscape, including lipids and lipid-like metabolites, amino acids, bile acids, and steroid-related metabolites.

**Conclusion:**

Our results demonstrate how the use of multi-omics to investigate complex host-microbiome interactions enable us to better evaluate the functional potential of probiotics compared to studies that only measure overall growth performance or that only characterise the microbial composition in intestinal environments.

Video Abstract

**Supplementary Information:**

The online version contains supplementary material available at 10.1186/s40168-021-01221-8.

## Background

Understanding how feed types and different biotic additives shape the intestinal microbiota and the biological interactions between host and bacteria is of paramount importance to continually boost sustainability of animal production. Pro- and prebiotics have often been considered to promote gut health and fish growth by decreasing the prevalence of intestinal pathogens and changing the synthesis of bacterial exo-metabolites [1] related to health and growth [2–4]. Furthermore, the application of biotic additives has also shown an increase of the absorptive surface for nutrient uptake, by increased density and length of host villi and microvilli [5, 6]. Bacteria able to increase production efficiency have been heavily investigated in terrestrial livestock, including both omnivorous species such as swine and poultry as well as herbivorous ruminants such as cattle and sheep. Highly investigated probiotic strains include *Lactobacillus*, *Pediococcus*, *Bifidobacterium*, and *Enterococcus* for ruminants [7–10], poultry [11, 12], and swine [13–15]. Further, an investigation of autochthonous probiotics suggested that bacteria that are naturally adapted to the gastrointestinal tract of a respective host species are more likely to colonise when provisioned as probiotics in the feed [16]. In light of this, detailed characterisation of host-microbe interactions, using more holistic approaches, is needed to better understand how we can actively optimise beneficial services provided by the gut microbiota for livestock [17–19].

Despite the projected growth and importance of aquaculture in feeding a growing human population [20], there is a vast knowledge gap on how the gut microbiota support their host fish [21], especially when compared to terrestrial livestock where intestinal metagenomes have been increasingly investigated [19, 22–25]. It is therefore of utmost importance to gain more specific knowledge of the functional potential of fish-related gut microbiomes. Some feed types containing probiotics have already been tested in relation to their effects on growth efficiency and disease resilience [26–29]. The probiotic strains considered for aquacultural species so far are often derived from terrestrial species and thus not known to be related to the fish gut environment [16]. Even though probiotic related lactic acid bacteria, including *Pediococcus, Leuconostoc*, and *Lactobacillus* previously has shown to be present in the gut microbiome of salmonids [5, 30], very little is still known about their function in the fish gut environment regarding nutrient utilisation and immune response modulations [30, 31]. Selection and testing strategies need to be optimised further for the following reasons; first, fish are exotherms, which means that temperature conditions can vary a lot compared to the more stable body temperatures of terrestrial animals. Second, many farmed fish are carnivorous and known to have highly divergent gut microbiota communities compared to their herbivorous terrestrial counterparts [32]. Thus, there is a need for more specific knowledge of the functional potential of the gut microbiota related to fish species. Nevertheless, the type of diet needed to produce fish in a sustainable manner is closer to that of terrestrial farmed animals than of wild fish, and as such, probiotic bacteria that could help the host optimise digestion and utilisation of non-fishmeal-based diets would be of great interest.

Several studies based on 16S rRNA gene profiling of the gut microbiome of salmonids have demonstrated that the gut microbiome is highly variable and influenced by a variety of external factors, but also that the intestinal environment is often characterised by low biodiversity of the gut microbiota, and domination by Proteobacteria (phylum), *Shewanella*, and *Mycoplasma* genera [33–35]. One study showed that an increase of insect-based proteins to rainbow trout (*Oncorhynchus mykiss*) increases the relative abundance of *Mycoplasma* in the gut microbiota [34]. Multiple studies have shown that abundance of *Mycoplasma* is positively associated with fish health and that it is an often dominant species in the gut microbiota of both wild and farmed salmonids [35–42]. However, very little is known about the function of this *Mycoplasma* and its metabolic interplay with its salmonid host. Furthermore, the underlying mechanisms of the discrepancy of *Mycoplasma*, being highly dominant or totally absent remains unknown, despite recent interests [42, 43]. To address these unknowns, we advocate for approaches to better understand the functional interactions between host fish and their associated microbiota species [2].

In this study, we use a non-targeted multi-omics approach to unravel the functional effects on the intestinal microbiota and intestinal metabolism when providing a probiotic to farmed rainbow trout. Specifically, we (I) investigate microbial shifts in the gut environment caused by probiotic and synbiotic additives using both 16S rRNA gene profiling and whole-genome metagenomics sequencing, (II) investigate the functional diversity of the gut microbiota, using metagenomics combined with high-resolution untargeted metabolomics, including both UHPLC-MS/MS and IC HR-MS/MS, (III) investigate the differential abundance of key growth and health-related metabolites in light of metagenomic profiles among fish reared on feed types with and without biotic additives, and (IV) use novel network-based approaches for chemical structural annotation to break down unknown metabolite classes and improve knowledge of unknown microbial metabolites, which may be correlated with higher performance in rainbow trout.

## Results

### Feed additives and nutrient utilisation

**Fig. 1:**
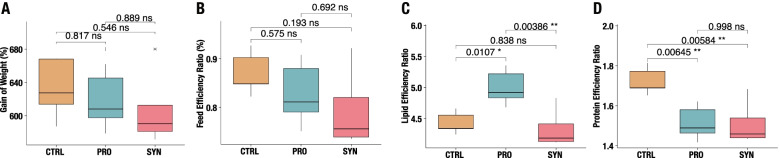
Performance test of feeding trial. Boxplots of growth performance attributes, including **A** Gain of Weight, **B** Feed Efficiency Ratio, **C** Lipid Efficiency Ratio, and **D** Protein Efficiency Ratio. Results from Tukey’s HSD tests for pairwise comparison between feeding groups are shown in brackets above boxplots. Feeding groups are visualised as orange for control feed (CTRL), blue for probiotic additive (PRO), and red for synbiotic additive (SYN)

Feed performance was evaluated based on bulk weights and counts of rainbow trout from five replicate tanks for each of the three feeding groups tested. Registration of feed administered to each tank, as well as near-infrared spectroscopically determined content of protein and lipid in each feed group was recorded (Fig. 1A–D).

Our findings indicate that nutrient-related phenotypes, like lipid and protein metabolism, of juvenile rainbow trout can be affected by functional diets. Overall, our analyses revealed no significant differences in percent weight gain (WG), feed conversion ratio (FCR), and the inverse of FCR, feed efficiency ratio (FER), among feeding groups (Fig. 1A, B). It should be noted that nutritional analysis of feed revealed a lower number of calories (MJ/Kg) and fat content (%) in PRO feed, and a lower amount of protein in the CTRL diet (Table 1). This may explain some of the observed differences in the performance data and complicates the further interpretations of feed efficiency indices (Fig. 1A–D and Supp. Figure S1).

**Table 1:** Overview of the feed types included and the main differences

Ingredient		Control samples (CTRL)	Probiotic samples (PRO)	Synbiotic samples (SYN)
Core feed composition	Fat content (%) (SD ± 1.21)	20.0	17.1	19.7
	Protein content (%) (SD ± 1.46)	52.8	57.4	55.1
	Calories (MJ/Kg) (SD ± 0.406)	22.37	21.55	22.45
Additives	*Pediococcus acidilactici* MA18/5M (1 × 10^6^ CFU/gram)	−	+	+
	Galacto-oligosaccharides (GOS) (1%)	−	−	+

Consequently, the lipid efficiency ratio (LER) suggests a significantly more effective conversion of feed lipids into biomass in the PRO group (F_(2,12)_ = 9.84, *p* = 0.0029) (Fig. 1C), indicating usage improved efficiency of lipid utilisation. Analysis of the protein efficiency ratio (PER) showed that the CTRL group had a significantly higher efficiency than the other feeding types (F_(2,12)_ = 9.88, *p* = 0.0029) 1D). An accurate LER and PER determination would require isoenergetic/proteinic diets and analysis of whole-body fat and protein rather than the bulk weight of the fish.

### Pro- and synbiotic additives are associated with a reduction in relative abundance of *Mycoplasma* throughout the rainbow trout intestine

**Fig. 2:**
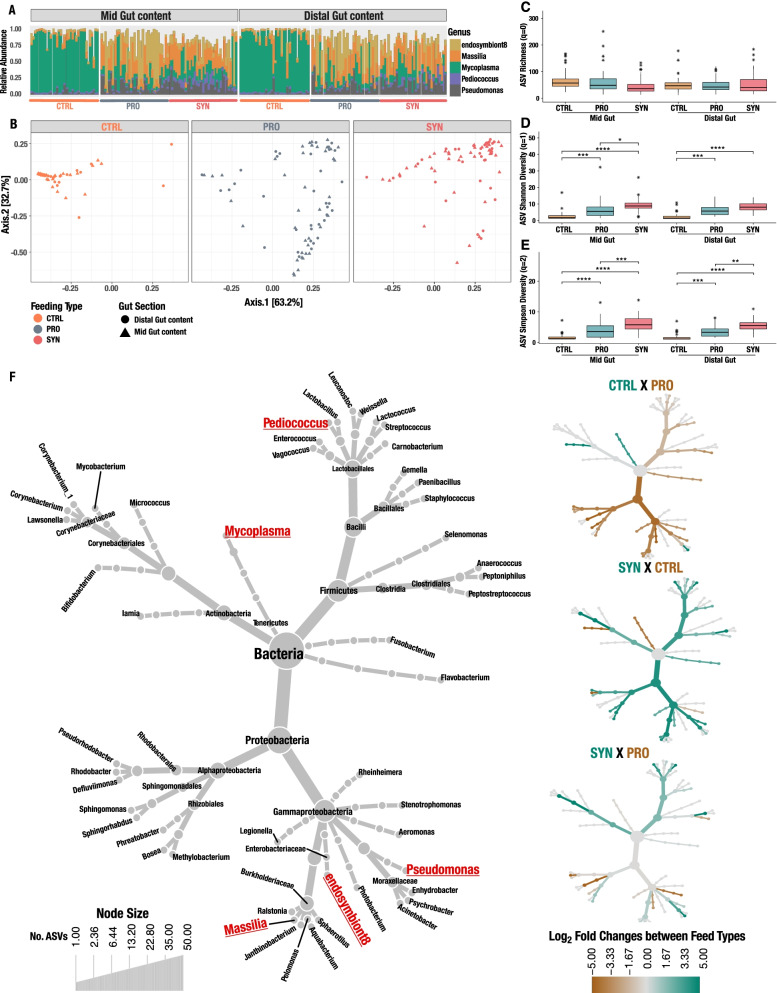
16S rRNA Gene profiling of the gut microbiome across feeding type and intestinal sections. **A** For the mid and distal gut sections separately, the barplots show relative abundance of high abundant bacteria defined as being present in more than 2% of all samples. **B** Principal Coordinates Analysis (PCoA) of the microbial composition in all samples and feeding groups using Unifrac distances. Distribution of individual samples for the three feeding groups are plotted for the same axes but shown in separate plots for visual purposes. Grouping of rainbow trout reared on different feeding types were visualised as orange for CTRL, blue for PRO, and red for SYN. Shapes indicate different intestinal sections, where the circle indicates samples isolated from distal gut content and triangles indicate samples isolated from mid gut content. **C**–**E** Boxplots of effective numbers of ASVs (Hill numbers) of microbiome across feeding types, including CTRL, PRO, and SYN. Order of effective numbers include **C **ASV Richness, *q* = 0, **D** ASV Shannon diversity (*q* = 1), **E** ASV Simpson diversity (*q* = 2). Significance code: [ns] > *α* = 0.05; [•] < *α* = 0.1; [*] < *α* = 0.05; [**] < *α* = 0.01; [***] < *α* = 0.001; [****] < *α* = 0.0001. **F** Heat Tree of species composition of the 50 most abundant ASVs throughout the gut combined with pairwise comparisons for the three feeding types, CTRL, PRO, and SYN. The grey tree on the lower left is a taxonomic reference for the smaller unlabelled trees. The most abundant genera from figure **A** are coloured in red and underlined. The colour of each taxon reflects differential abundance between the two groups being compared with colours determined by the log2 ratio of median proportions of reads observed in each feeding type. The Log_2_ changes were determined using a Wilcoxon rank-sum test followed by a Benjamini-Hochberg (FDR) correction for multiple comparisons. The size of nodes relates to the number of ASVs found within the given taxonomic group

During the experimental period, the rainbow trout were fed control feed (CTRL), probiotic feed (PRO), and synbiotic feed (SYN) (Table 1). Bacterial profiling of both the mid and distal intestinal content of a total of 120 juvenile rainbow trout (*n* = 40 fish per feed type), using the V3–V4 regions of the 16S rRNA gene, resulted in 382 amplicon sequence variants (ASVs). Filtering of low abundant ASV, by removal of ASV lower than 0.1% in mean relative abundance and removal of ASVs occuring in less than 2% of all samples, resulted in only six ASVs, indicating a low divers microbiota. Rarefaction curves were used to assess the saturation of recovered ASVs, suggesting an adequate sequence coverage for detecting present ASVs (Supp. Figure S2A-B). The five most abundant ASVs comprised 85.1% of the total number of microbial reads from rainbow trout in this trial, revealing a low intestinal microbiota diversity (mean effective ASV richness of 34.78 ± 15.8 Hill numbers). Taxonomy assignment revealed that the five most abundant ASVs included genera of *Mycoplasma*, *Pediococcus*, *Pseudomonas*, *Massilla*, and *endosymbiont8* (genus of *Enterobacteriaceae*). Bacterial profiling throughout the gut revealed significant changes in the microbial composition among different diet groups with fish from the CTRL group being dominated by *Mycoplasma*, compared to fish from the other two groups that were largely characterised by a higher relative abundance of *Massilla* and *endosymbiont8* (Fig. 2A). Further, the recovered *Pediococcus* ASV revealed an exact match with the administered probiotic strain of *P. acidilactici* MA18/5M.

Our analysis revealed a clear alteration of the microbiota because of feed type. A principal coordinates analysis (PCoA) revealed that 96.4% of the variance of the microbiota was explained by two principal components, and that the microbiota of CTRL clustered alone, whereas the microbiota of PRO and SYN clustered together (Fig. 2B). This pattern was repeated for both the mid and distal gut sections, but with no significant differences between the gut sections (Fig. 2B).

Diversity analysis based on Hill numbers [44, 45] revealed that richness was unaffected by gut section and feed (Fig. 2C), whereas we found a significantly higher microbial diversity in the microbiome in both the PRO and SYN groups compared to CTRL when taking relative abundance into account (Fig. 2D), and especially for highly abundant species (Fig. 2E).

Differential abundance analysis of the top 50 most abundant ASVs confirmed a significantly higher abundance of *Mycoplasma* in the CTRL group, indicating that feed additives may have a suppressing effect on the presence of *Mycoplasma* and *Bifidobacterium* (top and middle lateral panels in Fig. 2F). On the other hand, our data reveals an increase of the phylum Proteobacteria, the class of Clostridiales, the family of *Enterobacteriaceae*, and genera like *Pseudomonas*, *Massilia, Weissella*, and *Staphylococcus* in both PRO and SYN compared to CTRL (middle and bottom lateral panels in Fig. 2F)*.* The abundance of the probiotic *Pediococcus* ASV was significantly higher in the SYN group, compared to both the CTRL and the PRO groups, indicating that the usage of galacto-oligosaccharides (GOS) as a supplemental prebiotic in the SYN group did increase the abundance of *P. acidilactici* MA18/5M (bottom lateral panel in Fig. 2F).

### *Mycoplasma* abundance is associated with microbial pathways of known relevance for salmonid metabolism

A random subset of individuals from each feeding group was selected to investigate inherent microbes in the rainbow trout intestinal content. Deep sequencing of six individuals (from both mid and distal gut) was performed to get a minimal coverage, since the microbial biomass in intestinal samples was shown to be low as inferred from qPCR quantification of the V3–V4 regions of the 16S rRNA gene (Supp. Table S2.1). To cope with the high fraction of host DNA in the gut content samples, we aimed to deeply sequence these samples, resulting in more than 1.5 Tb of raw sequence data from the six individuals, including both mid and distal gut sections.

**Fig. 3:**
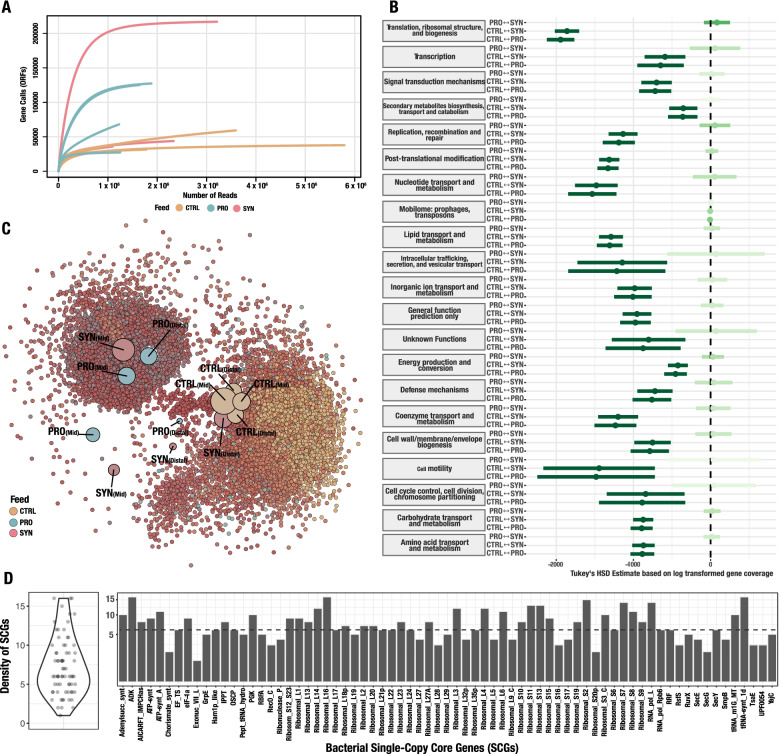
Metagenomic variation across feeding types, recovered from co-assembled samples. **A** Rarefaction curves of gene calls (ORFs) found because of sequencing depth. Curves are coloured according to feeding type, as indicated in legend. **B** Coefficient plot based on Tukey HSD of COG coverage between feeding types. Dots indicate Tukey’s HSD estimate and lines indicate confidence intervals. The shade of green indicates the relation between the estimate, confidence intervals, and null, meaning darkest green indicates significant estimates (p < 0.05). **C** Network of 17,354 COGs recovered from all samples. Big nodes indicate samples and small nodes indicate COGs. Distance between samples is based on similarity of samples. The size of sample nodes is based on the number of COGs connected to samples. The colour of COG nodes is based on the connectivity to sample nodes, where sample nodes are coloured as indicated in the legend. **D** Density plot and bar plot of bacterial SCGs are used to estimate the number of genomes in metagenomes. The dashed line in the bar plot indicates the highest density found of counted bacterial SCGs

Initial analysis of the metagenomic data revealed a saturation of open reading frames (ORFs) for all samples, indicating sufficient sequencing depth for the investigation of the rainbow trout metagenome (Fig. 3A). Subsequently, co-assembly of the 12 samples resulted in a recovery of 123,267 contigs, with a N50 on 3117 bp, and more than 238,000 ORFs. Annotation of ORFs, using cluster of orthologue genes (COGs), resulted in a recovery of 17,354 COGs. Comparison of mean gene coverage for COGs among feeding types, indicated a clear differentiation between CTRL and the feeding types including additives (PRO and SYN), with the CTRL group having the highest coverage of COGs for most categories (Fig. 3B).

Furthermore, investigation of COGs composition between samples and feeding types showed a high similarity among the four CTRL samples. The CTRL samples clustered away from both PRO and SYN, except for one SYN sample (Fig. 3C).

We also analysed bacterial single-copy core genes (SCG) in the co-assembled metagenome, using hidden Markov models (HMMs) to approximate the number of bacterial genomes present in the microbiome and to infer completeness and redundancy of recovered metagenome-assembled genomes (MAGs). Density analysis of SCG hits resulted in the highest density of six SCGs, indicating that no more than six distinct bacterial genomes were present in the metagenome of rainbow trout (Fig. 3D). This result further confirms the approximate number of highly abundant ASVs found, using 16S rRNA metabarcoding, characterising a low-diversity metagenome dominated by few species.

**Fig. 4:**
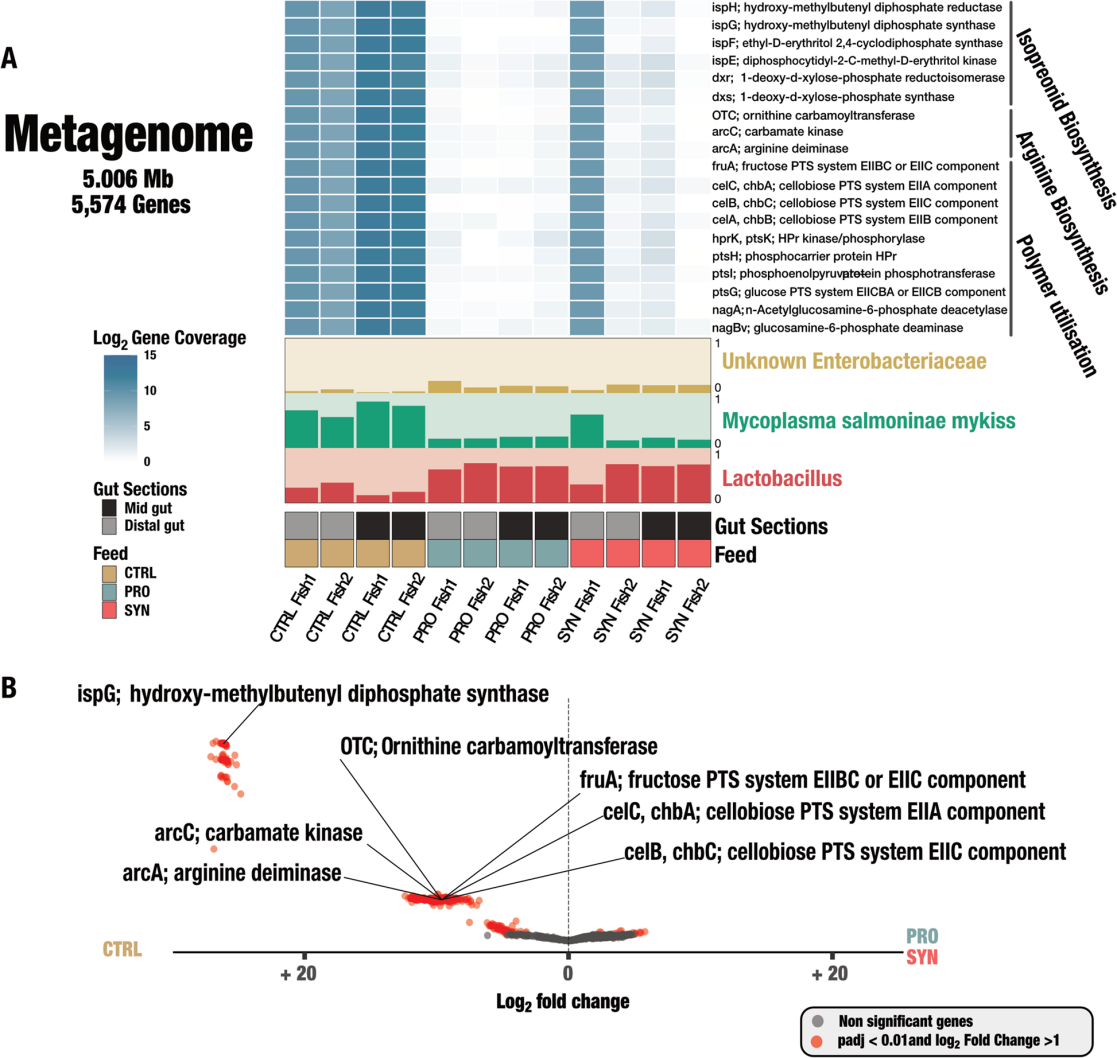
Genome-resolved metagenomics from gut microbiome across feeding type and intestinal sections. **A** Three metagenome-assembled genomes (MAGs) were resolved from the metagenome, consisting of 5.006 megabases (Mb) and 5574 non-redundant genes, which were visualised as yellow for *Unknown Enterobacteriaceae*, green for *Candidatus* Mycoplasma salmoninae mykiss, and red for *Unknown Lactobacillus*. Barplots indicate relative abundance of the two MAGs within each sample. Grouping of rainbow trout reared on different feeding types were visualised as orange for CTRL, blue for PRO, and red for SYN. Intestinal sections (gut sections) are coloured as black for mid gut and grey for distal gut. Heatmap visualises a series of genes of interest, which are related to isoprenoid biosynthesis, arginine biosynthesis, and polymer utilisation. Intensity of blue colour indicates log_10_ of coverage of genes across samples. **B** Volcano plot of differentially abundant metagenomic genes between samples from CTRL vs. PRO and SYN. Colouration of nodes indicate significance, whereas red nodes are significant genes after correction and have a log_2_ fold change (FC) > 1. Grey nodes are non-significantly differentiated genes between feeding types.

Generation of the curated MAGs and a MAG database resulted in a low diverse binned metagenome of 5.006 Mb, consisting of no more than 5,574 ORFs from two high-quality MAGs, with a completion higher than 80%, and no redundancy of SCGs. Subsequently, a medium-quality MAG with a completion of 52.11% and a low redundancy of 4.23 was also resolved (Fig. 4A and Table 2). We generated collector’s curves per sample to assess sequencing depth, and these indicated that saturation was near complete with our given level of sequencing (Supp. Fig. S3).

**Table 2:** Overview of MAGs resolved from the rainbow trout metagenome

MAGs	No. contigs	N50	Total Size (Mb)	GC Content (%)	Completion (%)	Redundancy (%)
Enterobacteriaceae	217	11,723	1.53	49.01	97.18	0
Mycoplasma	28	88,883	0.66	25.51	81.69	0
Lactobacillus	1637	1719	2.81	42.56	52.11	4.23

The draft genome for *Candidatus* Mycoplasma salmoninae mykiss (referred to as *Mycoplasma* in this study) has previously been reported [42], but here, we present the whole intestinal metagenome data retrieved from six rainbow trout, including both the mid and distal gut sections. *Mycoplasma* had an identical match with our previously found *Mycoplasma* ASV from the 16S rRNA gene profiling (Table 2). Further, a MAG of an unknown genus of *Enterobacteriaceae* corresponded to the presence of the *endosymbiont8* ASV, which we hypothesise to be the corresponding MAG for the *endosymbiont8* ASV (Table 2). Our analyses also resolve a MAG of an unknown *Lactobacillus* (Table 2). Lastly, short-read mapping of the metagenome revealed low levels of *Pediococcus acidilactici* MA18/5M genes present in rainbow trout from the PRO and SYN groups, indicating that the probiotic strain seems to be present at a low level in the intestinal content of fish exposed to the probiotic strain.

Our metagenomic analysis confirmed the bacterial composition found by 16S rRNA gene metabarcoding, where *Mycoplasma* was found to be highly dominant in CTRL and especially in the midgut, corresponding to 76.8–84.5% of all microbial reads in the midgut and between 56.4–68.7% of all microbial reads in the distal gut. This *Mycoplasma* dominance resulted in a Q2–Q3 mean coverage of 3667–5939X in the midgut samples and 317–847X in distal gut samples for CTRL, whereas the coverage of *Mycoplasma* in PRO and SYN was extremely low, except for one sample in SYN (Supp. Table S3.1). Both *Lactobacillus* and *Enterobacteriaceae* were found at higher relative abundance in fish from the PRO and SYN groups, as a reflection of a reduced *Mycoplasma* biomass (Fig. 4A). Interestingly, the coverage of *Lactobacillus* and *Enterobacteriaceae* were in general very low and ranged from 0.00 to 8.43X Q2–Q3 mean coverage across all samples for *Lactobacillus* and 0.00 to 5.16X Q2–Q3 mean coverage for *Enterobacteriaceae*, clearly indicating a low bacterial load even when abundance of *Mycoplasma* was reduced.

The functional potential of metagenomes also varied significantly among the feeding groups. Differential abundance analysis of the metagenome data revealed that 670 out of a total of 5574 non-redundant genes were significantly more abundant in CTRL (adjusted *p*-value < 0.05) (Fig. 2b, Supp. Table S3.2), including genes encoding for arginine biosynthesis pathway, such as *arcA*, *arcC*, and *otc* and genes associated with the cellobiose PTS system, referred to as cellulosome (Fig. 4B). Interestingly, we found terpenoid backbone synthesis-encoding genes, from the non-mevalonate (MEP) pathway, including *ispE*, *ispF*, *ispG*, and *ispH* to be enriched in the CTRL group (Fig. 4A, B). These MEP-related genes were all present in the *Mycoplasma* MAG, clearly indicating that the alterations of *Mycoplasma* abundance are the main driver of the observed metagenomic variation among feed groups. Surprisingly, we found that genes related to terpenoid backbone synthesis had a dramatically higher log_2_ fold change than the rest of the *Mycoplasma* MAG-related genes, which we hypothesise could reflect the presence of mobile genetic elements, such as genomic islands or plasmids, in *Mycoplasma*, though this is hard to detect using short read metagenomics technologies [46].

### Diet and *Mycoplasma* abundance are associated with the intestinal metabolism of rainbow trout

We aimed to generate a comprehensive representation of the intestinal metabolomic landscape including both ionic properties and polarity in our metabolic analysis. We included UHPLC-MS/MS and IC HR-MS/MS data generation [47, 48] resulting in a total of 22,222 mass spectral features with associated tandem mass spectrometric data, which we here use as a proxy for metabolites. Out of the 22,222 metabolites, 12,706 metabolites were generated from UHPLC-MS/MS and 9516 metabolites were generated from IC HR-MS/MS.

**Fig. 5:**
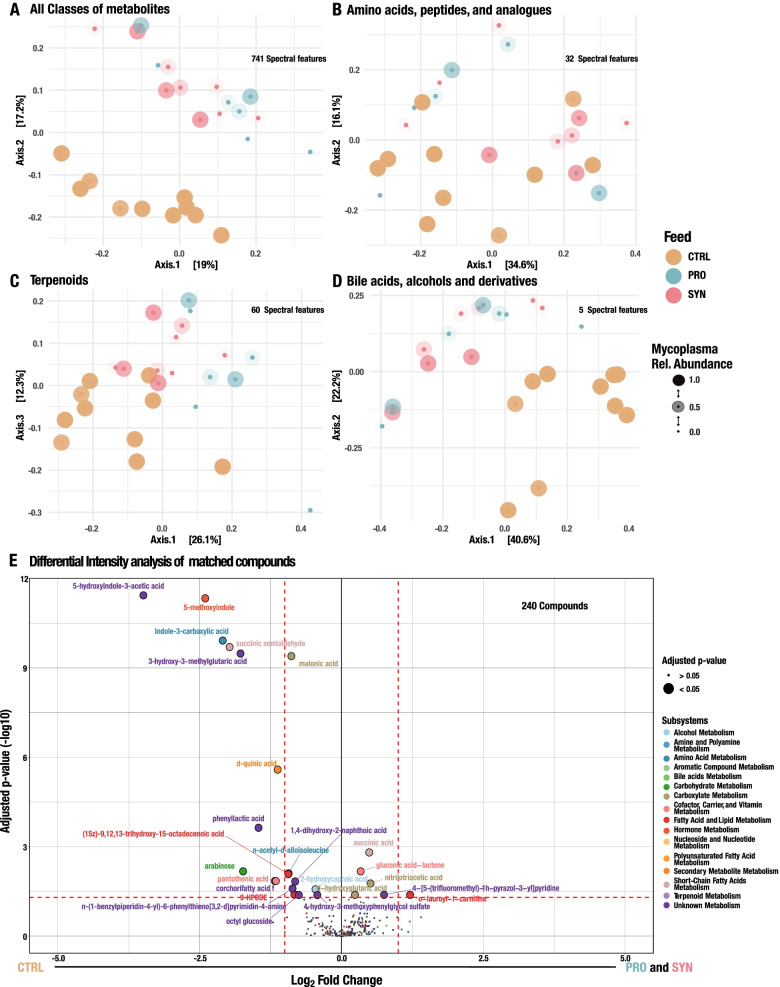
Composition of metabolic putative classes across feeding types and differentiation of known compounds. **A**–**D** Principal Coordinate Analysis (PCoA) of metabolites across all three feeding types based on 741 of the 9,723 metabolites, which is occurring in more than 50 of the 60 samples. Opacity of nodes is related to relative abundance of *Mycoplasma*. **A** All classes of metabolites, **B** Amino acids, peptides, and analogues, **C** Terpenoids, including monoterpenoid, diterpenoids, triterpenoids, and sesquiterpenoids, **D** Bile acids, alcohols, and derivatives. Grouping of rainbow trout reared on different feeding types were visualised as orange for CTRL, blue for PRO, and red for SYN. **E** Volcano plot of a differential intensity test performed between the CTRL feed against the two feed types with biotics additives. The test was based on 240 VSN normalised metabolites with a spectral match to known compounds. Metabolites with an adjusted *p*-value below 0.05 were significant. Size of nodes is dependent on adjusted *p*-value, where big nodes are significantly different between feeding types (*p*.adj < 0.05) and small nodes are not significant (*p*.adj > 0.05). The colour of nodes are dependent on related metabolic subsystems, which are specified in the legend

Using the molecular networks, we retrieved in silico annotated chemical classes for 7190 metabolites (56.59%) of UHPLC-MS/MS. Out of the 9723 metabolites, 741 metabolites were included in the study after filtering for false positives and zero elimination. Overall metabolic variations revealed a clear differentiation among the CTRL, PRO, and SYN (Fig. 5A). Specifically, we investigated metabolite classes putatively synthesised by enzymes encoded by genes found to be differentially abundant in the metagenomes (Fig. 3B and Fig. 4B). We found clear differentiations in compositions of putative metabolite subclasses between CTRL and the two other feed types. This differentiation of metabolites between feed types included amino acids, peptides, terpenoids, bile acids, alcohols, and derivatives (Fig. 5B–D). The composition of especially terpenoids did not only cluster samples based on feed type alone but also revealed some clustering of samples with a high relative abundance of *Mycoplasma* irrespective of feeding types (Fig. 5C).

For UHPLC-MS/MS, 419 (3.29%) could be matched to known compounds in the GNPS library. For IC HR-MS/MS, 282 (2.96%) could be matched to known compounds in the mzCloud database, which in total resulted in 240 known compounds after deduplication of isoforms of compounds and filtering (Supp. Table S3.4). Differential intensity analysis of the 240 known metabolites resulted in 25 differentially abundant metabolites, whereas 19 of these metabolites were more abundant in CTRL (Fig. 5E). These included pantothenic acid, indole-3-carboxylic acid, 5-methoxyindole, and 5-hydroxyindole-3-acetic acid, indicating a higher amount of vitamin B_5_ and degradation of tryptophan [49] in the gut of rainbow trout from the CTRL group. These differences indicate alterations of important immune-related metabolites among fish reared on the different feed types [49, 50]. Furthermore, we found an increase of succinic semialdehyde in CTRL, indicating butyrate-related short-chain fatty acid (SCFA) metabolism occurring in the gut of rainbow trout [51], which corresponds to previous findings that *Mycoplasma* dominates the microbiota of both wild and farmed Atlantic salmon [36]. SCFAs are known to be the end-products of dietary fibre fermentation by gut microbiota and have been suggested to be an essential nexus between microbiota and different host organ systems [52]. We found an increase of lauroyl-carnitine in PRO and SYN indicating fatty acid oxidation and thereby an increase in lipid metabolism. Furthermore, gluconic acid lactone was found increased in PRO and SYN, indicating induced sugar degradation, which we hypothesise is due to sugar formation by present *Lactobacillus* or *P. acidilactici* MA18/5M, which would make sense for SYN, where galacto-oligosaccharides were added to the feed (Fig. 4A and Fig. 5E).

Furthermore, differential intensity analysis of metabolites with no MS1 spectral hits revealed a total of 168 metabolites from UHPLC-MS/MS with a significantly different abundance between CTRL and the two other groups after FDR correction for multiple tests (adjusted *p*-value < 0.05) (Supp. Table S3.4). Furthermore, we found that 89 of the 168 metabolites were abundant in the CTRL group, whereas 79 of the metabolites were abundant in PRO or SYN. Metabolites more abundant in the CTRL group included the metabolite classes: prenol lipids, steroids and steroid derivatives, carboxylic acids and derivatives, and benzenes and substituted derivatives (Supp. Fig. S5, Supp. Table S3.5). These metabolite classes indicate a differentiation in steroid and terpenoid production in the intestinal environment. Especially prenol lipids, which include classes of terpenoids, were found to be highly affected by feed type and more abundant in the CTRL group thereby mimicking the differential abundance of *Mycoplasma* among feeding groups (Supp. Fig. S5). Further investigation of differentially abundant metabolites observed across feeding types confirmed our previous finding of the CTRL group having a distinct metabolomic landscape compared to the PRO and SYN groups (Fig. 5A–D, Supp. Fig. S6).

### Deciphering unknown metabolites associated with intestinal microbiota

To investigate association between specific metabolites and presence of microbes, we computed the correlation between the relative abundance of the ASVs per sample to the concentrations of a subset of the metabolites. We restricted our analysis to the 26 samples, which included both 16S rRNA gene profiling and metabolomics. The 26 samples included 10 fish from the CTRL group, nine from the PRO group, and seven SYN samples. Filtering out rare ASVs resulted in a total of six ASVs, while zero inflation of metabolites validated a total of 569 metabolites for this association analysis. Association tests between metabolite intensities and the relative abundances of ASVs revealed four metabolites that are significantly associated with the ASV abundances after Bonferroni correction [53] (Supp. Table S3.6). We investigated the top 25 most significantly bacterial associated metabolites (BAMs) post Bonferroni correction, using an enhanced molecular network [54–58] to infer these unknown metabolites in the intestinal metabolomic landscape of rainbow trout (Supp. Table S3.7).

**Fig. 6:**
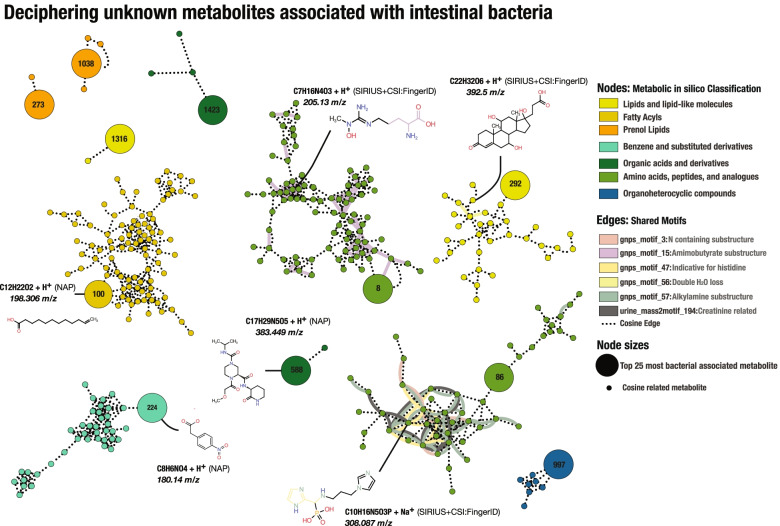
Molecular network showing bacterial associated metabolites and their molecular families with in silico annotation and hypothetical structures. Metabolomics-based molecular network of 11 bacterial associated metabolites (BAMs) and their molecular families coloured by putative chemical classes retrieved through a consensus of SIRIUS+CSI:FingerID and the GNPS based MolNetEnhancer workflow as indicated in the legend. A total of 350 metabolites were selected based on the bacterial association test and cosine relation to BAMs. Neighbour nodes of the significant nodes were co-selected to increase knowledge on chemical structural information of BAMs. Small nodes indicate metabolites in a network with bacterial associated metabolites. Edges are coloured according to shared motifs from MS2LDA and MotifDB databases or spectral similarity (cosine score > 0.7) as indicated in the legend. Unclassified molecular families were removed from the figure to increase clarity. Hypothetical structures suggested by in silico are shown and substructural motifs are highlighted with corresponding edge colours in the showed hypothetical structures

Network analysis of 350 metabolites, including the top 25 BAMs and their related molecular families, were used to decipher molecular structures of unknown metabolites (Fig. 6). MolNetEnhancer classified 92.5% of the metabolites, where 28.2% of the classifications were confirmed by SIRIUS+CSI:FingerID. Of the 25 BAMs and their related molecular families, we were able to classify 11 BAMs and their related molecular families. The molecular families included prenol lipids from terpenoid backbone synthesis, which were associated with intestinal bacteria. Furthermore, we found a molecular family of unknown lipids, with indications of water loss, indicating the formation of steroids, suggesting the formation of bacterial-related steroids in the intestinal environment (Fig. 6). Interestingly, we found BAMs related to networks of benzenoids, including putative stilbenes and phenylpropanones, indicating production of antibacterial BAMs in the intestinal environment of rainbow trout, which could target bacterial cell walls [59, 60].

Furthermore, we found BAMs in molecular families of fatty acyls with a relatively low mass charge, indicating conjugation of SCFAs. A molecular family of peptide structures, containing substructural motifs of SCFA-related aminobutyrate, indicating degradation, biosynthesis, or conjugation of an aminobutyrate-like peptide by bacteria in the intestinal environment. A network of putative peptides with BAMs revealed three shared substructural motifs between metabolites, including traces of histidine, alkylamine, and creatinine, indicating incorporation of ammonia derivatives into peptides by intestinal bacteria in rainbow trout (Fig. 6). These findings could potentially confirm our metagenomic observation of arginine biosynthesis, which includes the metabolism of ammonia-rich peptides.

## Discussion

We combine a rigorous, comparative feed trial with highly robust multi-omic data analysis, including deeply sequenced metagenomics, untargeted metabolomics, and bacterial 16S rRNA gene profiling of the intestinal compartment of rainbow trout. This multi-omics data set enabled us to first characterise rainbow trout-associated MAGs, including a recently described candidate *Mycoplasma* species, in relation to estimates of nutrient utilisation in rainbow trout. We then complemented the metagenome data with metabolomics insights on the variation in intestinal metabolites of rainbow trout. Together, we show how intake of functional feeds is associated with distinct microbiota functions and metabolic profiles in rainbow trout.

Our multi-omic investigation revealed significant effects of feed additives on the intestinal environment in rainbow trout. The growth trial showed an alteration of protein efficiency ratio and lipid efficiency ratio, when using probiotic or synbiotic feed diets, though we acknowledge that the differences in protein and fat content in these diets makes it difficult to attribute these changes to the additives in the diet only. The experimental setup was not designed to evaluate growth performance and that longer growth trials using diets with more similar nutritional content are needed. Therefore, the nutrient utilisation data should only be seen as preliminary associations rather than firm conclusions. Investigations of the microbiome using both genome-resolved metagenomics applications and SCGs revealed that only six distinct MAGs are present in the gut microbiome; here, we were able to recover three of these six putative MAGs. The metagenomic analysis was confirmed by 16S rRNA metabarcoding, where the top 5 most abundant ASVs recruited 85.1% of the metabarcoding data*.* Hence, our analysis revealed a low diverse microbiota, which were characterised by the dominance of *Mycoplasma*. *Mycoplasma* abundance was significantly reduced in fish feeding on diets with pro- or synbiotic additives. On the other hand, our data revealed an increase in *Pseudomonas*, *Enterobacteriaceae*, and *Massilia.* Based on these observations, we hypothesise that this probiotic induces a change in the microbiota, which can be mirrored in the meta-metabolism, potentially affecting both PER and LER. While such changes may be beneficial, it is interesting to note that previous studies have shown that *Mycoplasma* is consistently present in wild salmonid populations [36, 39, 42]. Further, neutral modelling comparing environmental and intestinal frequency distributions of *Mycoplasma* has previously suggested that Atlantic salmon-associated *Mycoplasma* are adapted to the colonisation of their hosts [61]. Together, our results add to previous findings and support the hypothesis that this salmonid-associated *Mycoplasma* genus might be a native symbiont to salmonid species.

Previous investigations of *P. acidilactici* MA18/5M in bigger rainbow trout have shown an increase of *Mycoplasma* in the gut microbiome, when using *P. acidilactici* MA18/5M as a feed additive, suggesting that these microbial alterations, induced by feed additives, are complex and further dependent on the age of the host, water salinity, diet composition of protein and fat, and dose of *P. acidilactici* MA18/5M [31, 62].

Functional insights from metagenomic analyses provided information on molecular pathways associated with the *Mycoplasma* MAG. The *Mycoplasma* MAG contains genes encoding enzymes involved in arginine biosynthesis, cellulosome, and terpenoid backbone synthesis, which is rather uncommon in *Mycoplasma* [1]. The induction of genes involved in terpenoid backbone synthesis in the transcriptome of salmonids has previously been reported to be positively correlated with FER [63], suggesting the importance of terpenoid backbone synthesis for growth metabolism. Therefore, we hypothesise that the terpenoid backbone synthesis encoded by the *Mycoplasma* genome might add a supplemental boost to the metabolism of their salmonid hosts. Subsequently, a recent study of *Mycoplasma* in Atlantic salmon correlated terpenoid production to increased pigmentation of salmon flesh, suggesting *Mycoplasma* is affecting the metabolism in Atlantic salmon [64]. Barring any unknown adverse effects of this dominant component of the rainbow trout intestinal tract, such metabolic contributions and apparent degree of host adaptation observed for the *Candidatus* Mycoplasma salmoninae, could suggest a fine-tuning of the performance of the trout from a hologenomics perspective [18].

Microbial contributions to the arginine biosynthesis pathway can affect host gut health. Microbial functions are likely to either (i) increase arginine analogous like ornithine and citrulline or (ii) decrease the amount of toxic ammonia in the intestine of ammonotelic teleosts such as salmonids through anabolic carbamate kinase activity during feeding [65–68]. Citrulline and ornithine have previously been found more efficient for amino acid uptake in rainbow trout [69, 70]. Ammonia reduction could have an impact on farmed fish, since they are often fed excessively, which suggests that *Mycoplasma* might serve as an advantageous gut symbiont that increases the tolerance of the host fish towards accumulated ammonia or suggests that *Mycoplasma* increase the availability of essential amino acids in the gut. Furthermore, we also found BAMs related to the incorporation of ammonia-rich moieties into putative peptides, further suggesting that bacteria in the intestinal environment are potentially detoxifying ammonia levels using several strategies [68].

High-resolution omics methods, like untargeted metabolomics, hold great potential for a more comprehensive understanding of the functional impacts provided by functional feed. Here, we found an increase of several compounds in fish fed live biotic additives, including the indication of increased beta-oxidation of long-chain fatty acids, by lauroyl−l−carnitine [71]. Together with the higher LER found in the PRO group, these findings clearly indicate metabolic changes because of probiotic feed additives.

The potential of using probiotics to improve growth and health in production animals has received immense attention across life sciences over the past decades. While there are successful examples of biotic additives, including a decrease of vertebral column compression syndrome [72], increased innate immune response in rainbow trout fingerlings [73], modulations of antiviral response [74], and an increase of absorptive surface in the host [5, 6] the underlying metabolic functions causing improved performance, or lack thereof, often remains unknown and especially so in the aquaculture field.

## Conclusions

Indeed, the microbiome of salmonids, and other commercially important fish species, have mainly been described using 16S rRNA gene profiling. These studies have led to several interesting and important hypotheses of host-microbiome interactions, but these hypotheses remain largely speculative as they are solely based on compositional data. Our results demonstrate how the use of multi-omics to investigate complex host-microbiome interactions enable us to better evaluate and explore the functional potential of probiotics compared to studies that only measure overall growth performance or that only characterise the microbial composition in intestinal environments. Furthermore, we demonstrate a more hologenomic approach to better understand complex host-microbe interactions in production animals based on a better functional understanding of intestinal microbiomes.

## Method and materials

### Rainbow trout and trial design

The sourcing of fish and rearing procedures used in this study have previously been described in a separate study [75]. Briefly, we obtained rainbow trout eggs from the AquaSearch FRESH strain (all-female, AquaSearch OVA, Billund, Denmark). Eggs were hatched and reared at the Bornholm Salmon Hatchery (Nexø, Denmark) that has a disease-free record and upon arrival, the eggs were disinfected using Desamar K30. Prior to experimental feeding, the fish were transported to the BioMar A/S research facilities (Hirtshals, Denmark). The initial mean weight was 4.3 g, when the fish arrived at the research facility at Hirtshals on January 30th, 2019. Each tank contained 80 fish at the start of the feeding phase. An experimental feeding trial was carried out over an 8-week period. Three experimental, proprietary feed formulations were selected (Table 1): (I) CTRL; a control feed without any pre- or probiotic additives, (II) PRO; control feed plus the commercial probiotic BACTOCELL with *Pediococcus acidilactici* MA18/5M, and (III) SYN; control feed with a synbiotic additive, consisting of BACTOCELL and galacto-oligosaccharides. To minimise tank effects we only sampled from one tank per feeding type.

To minimise sampling bias, all feeding types were double-blinded before sampling. This was upheld throughout the feed trials, sample processing, and analysis, and finally unblinded post analysis.

### Feeding and sample collection

For metabolomic investigation, 50 mg of gut content were sampled from the same region of the distal gut from 30 individuals, including 10 samples from each feeding type. Samples were immediately frozen on dry ice and subsequently transferred to a − 80 °C freezer within hours. For microbial profiling, a total of 120 rainbow trout, including 40 individuals from each feeding type, were sampled. Samples were taken from mid and distal gut sections at both time points by dissecting gut content from both sections, using sterile scalpels and tweezers, resulting in 240 samples for microbial profiling. Inoculation loops were used to ensure a normalised amount of 100 mg of gut content from each sample. All samples were preserved in SHIELD™, provided by Zymo Research, following the Zymo Research standard procedure. Weight, fork length, and qualitative comments regarding wounds were recorded for all individuals. All individuals were euthanised, according to the approved experimental guidelines, using Benzocaine in water prior to dissection, as detailed in a previous study [75].

### Feed performance analysis

Feed performance parameters were analysed for each group based on recorded bulk fish weight, numbers of individuals, and provided feed during the experimental feeding period for each of the five replicate tanks in each experimental feed group. Furthermore, the specific fat and protein content for each experimental feed was determined using near-infrared spectroscopy as part of internal quality control at BioMar [76]. Calculations of parameters were calculated according to previous study [75] (Supplementary information 3.3.6).

### Profiling the V3–V4 region of the bacterial 16S rRNA gene

DNA extractions for 16S rRNA gene profiling were carried out using Zymo Research Quick-DNA/RNA (Cat. D2131) following suppliers’ recommendation. Prior to analysis, all samples were randomised. Extracts were quality controlled for inhibitors and optimal PCR settings prior to metabarcoding. Two extraction negatives, two library negatives, and two PCR negatives were included for each plate. Metabarcoding was carried out by amplifying the V3-V4 region of the bacterial 16S rRNA gene, using the primers 341F (5′-CCTAYGGGRBGCASCAG-3′) and 806R (5′-GGACTACNNGGGTATCTAAT-3) [77] combined with unique forward and reverse 8-bp tags (Supp. Table S2.2). All amplifications were carried out in triplicates to lower the number cycles needed for PCR and to minimise procedural false positives [78]. PCR amplification were carried out, using 35 cycles. Library preparation was carried out using Illumina NEBNext® Ultra™ IIDNA Library Prep Kit. Amplicons were sequenced on an Illumina NovaSeq 6000 PE250bp to obtain 250bp paired-end reads aiming for a minimum 10,000 reads per PCR replica.

### Metagenomic data generation

Prior to analysis, all samples were randomised. Extraction of DNA for metagenomics was carried out using ZymoBiomics DNA miniPrep for a total of six rainbow trout, where two intestinal sections, including midgut and distal gut, were included, resulting in 12 samples (Supp. Table S2.3 for further details). Fragmentation of DNA to 400 bp was carried out, using Covaris M220 with microTUBE-50 AFA Fiber Screw-Cap. Samples were normalised to a 400-ng input for library preparation. Library preparation was based on single-tube library preparation for degraded DNA [79] (see Supp. information 2.2). Prior to the indexing of libraries, all libraries were analysed with quantitative PCR (qPCR) to estimate optimal cycle settings on a Mx3005P qPCR System (Agilent Technologies) (see Supp. Table S2.3).

Purified libraries were indexed and amplified for sequencing, using customised index primers for MGI-2000. Sequencing was carried out, using 150 PE chemistry on a MGI-2000 at BGI Europe. Data for five of the 12 samples were generated for a previous study [42]. The remaining 7 samples were processed, and the generated data was analysed for this study and has not been published prior to this study (see Supp. Table S2.4 for further details).

### Metabolomic extraction and preparation

A subset of ten samples from each of the three feeding types were selected according to Fulton's condition factor (five random samples below *K* = 2, and five samples above *K* = 2), resulting in a total sample size of 30 samples. To minimise batch effects all samples were randomised prior to any laboratory processing. Samples were homogenised in 100% methanol (MeOH) in a 1:10 sample:solvent ratio. Homogenisation was carried out in an OMNI Bead Ruptor 24, using dry ice to keep homogenised samples around 0 °C to minimise degradation of metabolites during homogenisation. Six procedural blanks were included in homogenisation. A volume of 100 μl of all samples was collected into quality control samples (QC samples) used for normalisation to enhance detection of metabolites;; all samples were purified after homogenisation, using solid-phase extraction (SPE), 2 mg/HRP-microSPE. The SPE was carried out conditioning with 200 μL 100% MeOH and washing with 200 μL 0.1% formic acid. Samples were eluted with 2 × 100 μL MeOH. Samples were concentrated using SpeedVac (ThermoFisher Scientific) and resuspended in 100 μL 5% MeOH. To correct for biases related to injection order, samples were divided into two replicates and ordered in an antiparallel order prior to nano-flow ultra-high pressure liquid chromatography-tandem high-resolution mass spectrometry analysis. Metabolites were detected and quantified using a Q Exactive™ HF Hybrid Quadrupole-Orbitrap™ Mass Spectrometer (ThermoFisher Scientific) operated in positive ion data-dependent acquisition mode (Supp. Information 2.3). Sample extracts were also analysed for more polar metabolites with ion-exchange chromatography hyphenated to a Q Exactive™ HF Hybrid Quadrupole-Orbitrap™ Mass Spectrometer (ThermoFisher Scientific) operated in negative ion data-dependent acquisition mode. A Dionex IonPac AS19-4 μm (2 × 250 mm) column was fitted with a Dionex AG19-4 μm (2 × 50 mm) Guard and connected to an ADRS 600 (2 mm) suppressor. Potassium hydroxide was used as an eluent.

### Bacterial 16S rRNA gene profiling of taxonomy and compositional analysis

Raw sequence data were quality controlled, using FastQC/v0.11.8 [80] to remove low-quality reads. Demultiplexing and removal of adaptors and low-quality reads were done with AdapterRemoval/v2.2.4 [81], with a base quality threshold of 30 and a minimum read length of 50bp. Microbial 16S data were further filtered with a maximum EE score or 2 for forwards and reverse reads. Reads were trimmed according to the error rate algorithm applied in DADA2 [82]. ASVs were clustered, using the clustering algorithm implemented in DADA2. Taxonomy was assigned through DADA2 using Silva/v138, using the implemented classification algorithm in DADA2 and the Silva database training set. Post clustering algorithms were applied to minimise false positives using LULU [83], and subsequently, contaminations were removed from samples, using decontam [84]. Lastly, ASVs were agglomerated by genera to minimise noise. Composition analyses were carried out using phyloseq [85] and differential abundance analyses across feeding groups were carried out using metacoder using the Wilcoxon rank-sum test and FDR correction for multiple comparisons [86]. Diversity analysis of the gut microbiota across feeding types was carried out applying Hill numbers, using hilldiv [44, 45, 87].

### Metagenomic bioinformatics: filtering, assembly, binning, refinement, and functional analysis

A subset of the data generated for this study were used for a separate, comparative study. Processing of data is presented in Rasmussen et al. 2021 [42]. Raw sequence reads were quality controlled, using FastQC/v0.11.8 [80] to assess filtering and quality steps. Removal of adapters and low-quality reads were done with AdapterRemoval/v2.2.4 [81], with a quality base of 30 and a minimum length of 50 bp. Duplicates were removed, and reads were re-paired to remove singletons, using bbmap/v.38.35 [88]. In order to increase assembly efficiency by reducing eukaryotic contaminants, data were filtered for the phiX174 genome, human (HG19) genome, and the rainbow trout (Omyk_1.0), using minimap [89]. Filtered data were both single assembled and co-assembled, using MegaHIT/v.1.1.1 [90] with a minimal length of 1000 bp per scaffold, using meta-sensitive flag for metagenomic purpose and assembled contigs were quality controlled with Quast/v.5.02 [91]. To increase effective binning, we used the anvi’o pipeline [92], as described in a previous study [42]. The relative abundance of each MAG was calculated based on percentage read recruitment across all samples from the specific host. Anvi’o was used to profile the scaffolds using Prodigal/v2.6.3 [93] with default parameters to identify genes and HMMER/v.3.355 matching archaeal [94], Protista (based on http://merenlab.org/delmont-euk-scgs) and bacterial [94] single-copy core gene collections. Also, ribosomal RNA-based HMMs were identified based on https://github.com/tseemann/barrnap. Completeness and redundancy of MAGs were calculated based on SCGs in anvi’o databases. Predicted gene functions were annotated using Pfam [95], COG [96], and KEGG [97]. Analyses of COG coverage in the whole metagenome were carried out, using Tukey’s HSD. The composition of COGs across feeding types and samples was carried out using a network-based approach by applying Gephi/v0.9.2 [98]. Functional networks were generated using the Force Atlas 2 algorithm to connect COG functions and samples using 20,000 iterations. Differential abundance analysis of all genes recovered from MAGs was carried out, using generalised linear models (GLMs) in DESeq2 [99] in R. We applied a Benjamini-Hochberg false discovery rate correction to *p*-values to account for multiple tests [100].

### Metabolomic annotation and metabolite substructural analysis

ThermoFisher Scientific UHPLC-Orbitrap-MS/MS RAW files were converted into mzML files using Proteo Wizard [101]. A molecular network was created using the classical molecular networking workflow https://ccms-ucsd.github.io/GNPSDocumentation on the Global Natural Product Social Molecular Networking (GNPS) platform http://gnps.ucsd.edu [58, 102].

In order to enhance the identification of unknown metabolites, unsupervised substructures were discovered using MS2LDA [57, 103], and MS2 spectra were annotated in silico using network annotation propagation (NAP) [103]. Furthermore, peptidic natural products (PNPs) were annotated in silico, using DEREPLICATOR [104]. Chemical classes were retrieved for all GNPS library hits and in silico structures using ClassyFire [54]. Finally, all structural annotations were combined within one network using MolNetEnhancer [104]. Further annotation of metabolites was carried out, using MetDNA [105].

The obtained IC HRMS/MS data processed using Compound Discoverer 3.2.0.421 (Thermo Scientific). The optimised workflow performed retention time alignment, compound identification (detailed in Supp. Table S3.3).

### Metabolomic compositional analysis of UHPLC-Orbitrap-MS and differential intensity analysis of UHPLC-Orbitrap-MS/MS and IC HR-MS/MS known metabolites

Quality assessment of initial data from UHPLC-Orbitrap-MS/MS was carried through PCoA plotting to ensure proper quality of quality pools, samples, and procedural blanks (Supp. Fig. S4). To minimise false positive metabolites, we removed high abundant (metabolites above 5 × 10^6^ Summed Precursor Ion Intensities) metabolites present in procedural blanks, which resulted in 9,863 metabolites. Metabolites present in > 50 of the 60 samples were kept minimising zero inflation in metabolomic data, resulting in 741 metabolites. Procedural replicates were average between individuals for group comparisons, as the replicates did not seem to affect the variation between groups (Supp. Fig. S4). PCoA of metabolites were carried out, using phyloseq [105]. We carried out a PCoA based on jaccard distances to minimise biases related to the relative abundance of precursor intensities of unknown metabolites generated from UHPLC-Orbitrap-MS/MS.

Detected compounds from UHPLC-Orbitrap-MS/MS and IC HR-MS/MS were imputed to minimise zero inflation and normalised with variance stabilising normalisation, using MetaboDiff [106]. Technical replicates were averaged. Precursor intensities of isomers and conjugated compounds were summed (detailed in Supp. information 3.3). Differential intensity analysis was carried using MetaboDiff (Supp. Table 3.4). To increase biological inference of compounds, we analysed compounds, using MetaCyc [107] (detailed in Supp. information 3.3).

### Association between bacterial ASVs abundances and metabolites

We restricted our analysis to the 26 samples that had their microbiome profiled using the 16S amplicon sequencing and had metabolites measured. Of these 26 samples, including 10 from CTRL, 9 from PRO, and the remaining 7 from the SYN group.

We filtered out the rare ASVs and retained only the six most abundant ASVs to restrict our analyses to only the most relevant ASVs, where we would have statistical power to detect associations. Metabolite data were filtered and normalised (detailed in Supp. information 3.3). To measure the putative effect of ASVs on metabolite relative abundances, for each metabolite, we used a stepwise regression procedure, using R-package MASS, where we started with a linear model where the abundances of all six ASVs were used as explanatory variables (detailed in Supp. information 3.3). The final model was selected when no ASVs could be removed without significant reduction in the explanatory power of the model, and no ASVs could be added with significant improvement of fit.

Finally, note that we did not include feed type as an explanatory variable for the metabolite relative abundances, since the feed type was highly correlated with the ASV abundance information. Specifically, the abundance of *Mycoplasma* was indicative of control vs. non-control feed type. Thus, given the modest sample sizes in this study, we decide to focus our test on the putative effects of ASV abundance on metabolite relative abundances.

For each metabolite, we tested the final model obtained from the stepwise procedure using a *F*-statistic to test the proportion of variance explained by the chosen ASVs, we used the Bonferroni correction for significance adjustment (detailed in Supp. information 3.3).

### Metabolomic network analysis of bacterial-associated metabolites and substructural analysis

Networks for selected Bacterial associated metabolites (BAMs) were visualised using Cytoscape/v3.8.0 [108]. Annotations of BAMs were carried out as detailed for UHPLC-MS/MS data and with a combination of GNPS network, MolNetEnhancer, and SIRIUS4 with CSI:FingerID [109, 110] (detailed Supp. information 3.3). Information of nodes within the metabolic networks is detailed in Supp. (Supp. Table S3.7).

### Statistical analysis

All statistics were conducted using R (version 3.6.1) and Python (version 3.7.4).

## Supplementary Information

**Additional file 1** Supplementary information.

**Additional file 2**

**Additional file 3**

## Data Availability

Summary of metagenome is publicly available upon acceptance at: 10.6084/m9.figshare.13193846. Anvi’o database for metagenomics analysis is publicly available upon acceptance at: 10.6084/m9.figshare.17142428. The data generated for 16S rRNA gene profiling of bacteria are available in the ENA repository with project accession number PRJEB48695. The raw metagenomic dataset generated during the current study will be available in the ENA repository with project accession numbers PRJEB40990. The metabolomics datasets generated and analysed during the current study are available in the MassIVE repository, using accession number MSV000084364, ftp://massive.ucsd.edu/MSV000084364/. Any code used for the study used to generate results that are reported in the paper and central to its main claims is available at: https://www.github.com/JacobAgerbo/Multi_Omic_Rainbow_Trout.
