# Supplementary material for: A multi-omics approach unravels metagenomic and metabolic alterations of a probiotic and synbiotic additive in rainbow trout (Oncorhynchus mykiss)
Source: Microbiome. 2022 Jan 30;10:21. doi: 10.1186/s40168-021-01221-8 (PMC8802455; doi:10.1186/s40168-021-01221-8)
Supplement: Supplementary file 2 — Additional file 1 Supplementary information. [file 40168_2021_1221_MOESM2_ESM.docx]

Supplementary information

# 1 Trial and Sampling

## 1.1 Fish

Rainbow trout from commercially available stock (AquaSearch FRESH, 100% females, AquaSearch ova Aps, Billund, Denmark) were used for the three trials included in this study. Eggs were hatched and reared under pathogen-free conditions at AquaBaltic (Nexø, Denmark). Prior to each respective trial, fish were transferred to experimental facilities at the BioMar A/S Research Centre (North Sea Science Park, Hirtshals, Denmark). Upon arrival, fish were divided into groups of 30–100 fish per 170 l tanks (trial dependent) and kept at a water temperature of 14 ± 0.2 °C. A 14 h light / 10 h dark cycle was maintained during all trials. A two-week acclimatization period, during which all fish were fed basic commercial feed pellets suitable for each respective fish size (BioMar A/S, Denmark) corresponding to 1.5% body weight per day using band-feeders (12h), was allowed prior to the trial.

## 1.2 Feeding and Sample collection

Trials of feeding type were carried out over eight weeks. Three feeding types were selected, I) a control feed without any additives, II) control feed plus BACTOCELL (a commercial probiotic for fish and shrimps with Pediococcus acidilactici MA18/5M, and III) control feed with synbiotic additives, consisting of BACTOCELL and Galacto oligosaccharides (GOS). In order to minimise sampling bias were all feeding types blinded before sampling. Blindfolding was kept until after analysis of results.

Sample collection was carried out over two sampling periods: four weeks after trial start and eight weeks after trial start. First week of sampling included n=20 per feeding type and last sampling included n=40 per feeding type. Approximately 50mg of gut content were sampled from the same region of the distal gut in each individual. Samples were immediately frozen at -80°C, using dry ice (CO2). For allochthonous investigation of microbiome, using metagenomic and 16S profiling approaches, samples were taken in the mid- and distal gut section at both time points, by dissecting out gut content from both sections, using sterile scalpels and tweezers. For investigation of metabolomic and meta-metabolomic profiling samples were taken in distal gut, using the same sampling approach. Inoculation needles were used to ensure a semi normalised amount of gut content from each sample. All samples were preserved in SHIELD™, provided by Zymo Research, following Zymo Research standard procedure. Weight and length were notified for each individual. All individuals were euthanised, according to the approved experimental guidelines, using Benzocaine in water bath prior dissection.

# 2 Laboratory Procedures

## 2.1 16S rRNA gene profiling of bacteria

DNA extractions for 16S profiling were carried out using Zymo Research Quick-DNA/RNA (Cat. D2131) following suppliers’ recommendation, using SHIELD (Zymo Research) as preservation buffer.

Real‐time PCR (qPCR) was performed on all extracts prior to metabarcoding, to optimize the subsequent metabarcoding process. Specifically, all DNA extracts were pre-screened using) with each primer sets to (a) screen for contamination in extraction negatives, (b) identify the potential presence of PCR inhibitors, and (c) optimize the cycles needed for metabarcoding PCRs. qPCR was performed in 20 μl reactions containing either 1 µl 2:1, 1:1, or 1:10 DNA template, and 1X AccuPrime SuperMix II (Invitrogen), 6.5µl ddH_2_0, 0.1 µM forward, and reverse primers, and 1 μl of SYBR Green/ROX solution (Invitrogen). qPCR amplifications were performed on an Mx3005 qPCR machine (Agilent Technologies) with the following cycling conditions: 95°C for 10 min, followed by 40 cycles of 95°C for 15 s, 55°C for 20 s, and 72°C for 40 min.

Metabarcoding PCR was performed in 20 µl reactions, consisting of 1 µl of 1:1 DNA, and 1X AccuPrime SuperMix II (Invitrogen), 6.5µl ddH_2_0, 0.1 µM forward, and reverse primers. PCR amplifications were carried out using an Applied Biosystems 2,720 Thermal Cycler with the following conditions: 95°C for 5 min, followed by 38 cycles of 15 s at 95°C, 20 s at 55°C, and 40 s at 72°C, followed by 72°C for 10 min. PCR products were visualized using gel electrophoresis (GE). PCR replicates were pooled into a single pool based on gel intensity.

All amplifications were carried out in triplicates to minimise procedural false positives(Alberdi et al. 2018). Library preparation was carried out using Illumina NEBNext® Ultra™ IIDNA Library Prep Kit. Amplicons were sequenced on an Illumina NovaSeq 6000 PE250bp to obtain 250bp paired end reads aiming for a minimum 10,000 reads per PCR replica.

**Supplementary Table S2.1 PCR cycles from real-time quantification (qPCR) of bacterial 16S rRNA gene based on a subset of samples used in this study.**

| CTRL.Feed 1 | F5 | 6 | 1:1 | 22,42 |
| --- | --- | --- | --- | --- |
| CTRL.Feed 1 | F11 | 6 | 1:10 | 21,98 |
| CTRL.Feed 2 | G5 | 6 | 1:1 | 27,52 |
| CTRL.Feed 2 | G11 | 6 | 1:10 | 22,32 |
| CTRL.Feed 3 | H5 | 6 | 1:1 | 28,61 |
| CTRL.Feed 3 | H11 | 6 | 1:10 | 23,41 |
| CTRL.T01.F01.S4 | D2 | 5 | 2:1 | 26,77 |
| CTRL.T01.F01.S4 | D6 | 5 | 1:1 | 33,58 |
| CTRL.T01.F01.S4 | D10 | 5 | 1:10 | 34,09 |
| CTRL.T01.F02.S3 | A4 | 5 | 2:1 | 42,5 |
| CTRL.T01.F02.S3 | A8 | 5 | 1:1 | 36,76 |
| CTRL.T01.F02.S3 | A12 | 5 | 1:10 | 39,52 |
| CTRL.T01.F02.S4 | C2 | 6 | 2:1 | 25,96 |
| CTRL.T01.F02.S4 | C8 | 6 | 1:1 | 28,55 |
| CTRL.T01.F03.S3 | G4 | 3 | 2:1 | 26,65 |
| CTRL.T01.F03.S3 | G8 | 3 | 1:1 | 39,36 |
| CTRL.T01.F03.S3 | G12 | 3 | 1:10 | 34,7 |
| CTRL.T01.F03.S4 | H1 | 3 | 2:1 | 32,4 |
| CTRL.T01.F03.S4 | H5 | 3 | 1:1 | 36,34 |
| CTRL.T01.F03.S4 | H9 | 3 | 1:10 | 32,68 |
| CTRL.T01.F06.S3 | D4 | 3 | 2:1 | 35,14 |
| CTRL.T01.F06.S3 | D8 | 3 | 1:1 | 32,96 |
| CTRL.T01.F06.S3 | D12 | 3 | 1:10 | 37,56 |
| CTRL.T01.F10.S4 | D4 | 5 | 2:1 | 35,47 |
| CTRL.T01.F10.S4 | D8 | 5 | 1:1 | 35,57 |
| CTRL.T01.F10.S4 | D12 | 5 | 1:10 | 34,7 |
| CTRL.T01.F12.S4 | D3 | 6 | 2:1 | 35,73 |
| CTRL.T01.F12.S4 | D9 | 6 | 1:10 | 26,43 |
| CTRL.T01.F13.S4 | H4 | 1 | 2:1 | 31,1 |
| CTRL.T01.F13.S4 | H8 | 1 | 1:1 | 34,24 |
| CTRL.T01.F15.S3 | B3 | 3 | 2:1 | 29,7 |
| CTRL.T01.F15.S3 | B7 | 3 | 1:1 | 40 |
| CTRL.T01.F15.S3 | B11 | 3 | 1:10 | 32,67 |
| CTRL.T01.F15.S4 | B3 | 1 | 2:1 | 31,63 |
| CTRL.T01.F15.S4 | B7 | 1 | 1:1 | 34,44 |
| CTRL.T01.F15.S4 | B11 | 1 | 1:10 | 34,86 |
| CTRL.T01.F18.S4 | D1 | 1 | 2:1 | 34,08 |
| CTRL.T01.F18.S4 | D5 | 1 | 1:1 | 36 |
| CTRL.T01.F18.S4 | D9 | 1 | 1:10 | 34,99 |
| CTRL.T01.F27.S4 | E2 | 6 | 2:1 | 24,03 |
| CTRL.T01.F27.S4 | E8 | 6 | 1:1 | 26,22 |
| CTRL.T01.F28.S4 | F2 | 1 | 2:1 | 33,84 |
| CTRL.T01.F28.S4 | F6 | 1 | 1:1 | 33,87 |
| CTRL.T01.F28.S4 | F10 | 1 | 1:10 | 33,65 |
| CTRL.T01.F30.S3 | C3 | 6 | 2:1 | 34,47 |
| CTRL.T01.F30.S3 | C9 | 6 | 1:10 | 31,06 |
| CTRL.T01.F33.S4 | D4 | 6 | 2:1 | 26,52 |
| CTRL.T01.F33.S4 | D10 | 6 | 1:10 | 27,64 |
| CTRL.T01.F38.S3 | C1 | 6 | 2:1 | 26,65 |
| CTRL.T01.F38.S3 | C7 | 6 | 1:1 | 29,68 |
| CTRL.T01.F38.S4 | E4 | 6 | 2:1 | 28,34 |
| CTRL.T01.F38.S4 | E10 | 6 | 1:10 | 25,87 |
| Extraction Neg | F2 | 3 | 2:1 | 40 |
| Extraction Neg | F6 | 3 | 1:1 | 37,06 |
| Extraction Neg | F10 | 3 | 1:10 | 37,8 |
| Extraction Neg | G4 | 6 | 2:1 | 38,3 |
| Extraction Neg | H4 | 6 | 2:1 | 39,7 |
| Extraction Neg | D6 | 6 | 1:1 | 35,6 |
| Extraction Neg | H6 | 6 | 1:1 | 38,4 |
| Extraction Neg | G10 | 6 | 1:10 | 40 |
| Extraction Neg | H10 | 6 | 1:10 | 36,7 |
| Extraction Neg | D12 | 6 | 1:10 | 37,04 |
| Extraction Neg | H12 | 6 | 1:10 | 36,03 |
| Library Blank | A3 | 1 | 2:1 | 40 |
| Library Blank | F3 | 1 | 2:1 | 37,04 |
| Library Blank | A4 | 1 | 2:1 | 40 |
| Library Blank | A7 | 1 | 1:1 | 40 |
| Library Blank | F7 | 1 | 1:1 | 40 |
| Library Blank | A8 | 1 | 1:1 | 36,47 |
| Library Blank | A11 | 1 | 1:10 | 36,92 |
| Library Blank | F11 | 1 | 1:10 | 40 |
| Library Blank | A12 | 1 | 1:10 | 40 |
| Library Blank | F3 | 3 | 2:1 | 40 |
| Library Blank | F7 | 3 | 1:1 | 37,06 |
| Library Blank | F11 | 3 | 1:10 | 40 |
| Library Blank | F4 | 6 | 2:1 | 35,19 |
| Library Blank | F10 | 6 | 1:10 | 38,54 |
| PCR Blank | H2 | 6 | 2:1 | 40 |
| PCR Blank | H8 | 6 | 1:1 | 40 |
| PCR.Blk.5 | G2 | 1 | 2:1 | 40 |
| PCR.Blk.5 | G6 | 1 | 1:1 | 40 |
| PCR.Blk.5 | G10 | 1 | 1:10 | 40 |
| PRO.Feed 1 | A6 | 6 | 1:1 | 24,13 |
| PRO.Feed 1 | A12 | 6 | 1:10 | 26,34 |
| PRO.Feed 2 | B6 | 6 | 1:1 | 25,15 |
| PRO.Feed 2 | B12 | 6 | 1:10 | 26,86 |
| PRO.Feed 3 | C6 | 6 | 1:1 | 24,83 |
| PRO.Feed 3 | C12 | 6 | 1:10 | 29,06 |
| PRO.T01.F02.S4 | D2 | 3 | 2:1 | 34,78 |
| PRO.T01.F02.S4 | D6 | 3 | 1:1 | 33,17 |
| PRO.T01.F02.S4 | D10 | 3 | 1:10 | 34 |
| PRO.T01.F04.S4 | B4 | 1 | 2:1 | 34,59 |
| PRO.T01.F04.S4 | B8 | 1 | 1:1 | 34,43 |
| PRO.T01.F04.S4 | B12 | 1 | 1:10 | 32,01 |
| PRO.T01.F05.S3 | C1 | 1 | 2:1 | 35,47 |
| PRO.T01.F05.S3 | C5 | 1 | 1:1 | 36,47 |
| PRO.T01.F05.S3 | C9 | 1 | 1:10 | 34,52 |
| PRO.T01.F05.S4 | F1 | 6 | 2:1 | 33,08 |
| PRO.T01.F05.S4 | F7 | 6 | 1:1 | 33,35 |
| PRO.T01.F06.S4 | H3 | 5 | 2:1 | 33,97 |
| PRO.T01.F06.S4 | H7 | 5 | 1:1 | 40 |
| PRO.T01.F06.S4 | H11 | 5 | 1:10 | 34,88 |
| PRO.T01.F11.S4 | F4 | 1 | 2:1 | 31,87 |
| PRO.T01.F11.S4 | F8 | 1 | 1:1 | 35,99 |
| PRO.T01.F11.S4 | F12 | 1 | 1:10 | 40 |
| PRO.T01.F17.S3 | C3 | 5 | 2:1 | 39,92 |
| PRO.T01.F17.S3 | C7 | 5 | 1:1 | 36,36 |
| PRO.T01.F17.S3 | C11 | 5 | 1:10 | 35,55 |
| PRO.T01.F20.S4 | F3 | 5 | 2:1 | 34,98 |
| PRO.T01.F20.S4 | F7 | 5 | 1:1 | 40 |
| PRO.T01.F20.S4 | F11 | 5 | 1:10 | 37,28 |
| PRO.T01.F21.S4 | G4 | 1 | 2:1 | 35,9 |
| PRO.T01.F21.S4 | G8 | 1 | 1:1 | 33,74 |
| PRO.T01.F22.S3 | E1 | 6 | 2:1 | 31,82 |
| PRO.T01.F22.S3 | E7 | 6 | 1:1 | 33,75 |
| PRO.T01.F22.S4 | D3 | 1 | 2:1 | 35,25 |
| PRO.T01.F22.S4 | D7 | 1 | 1:1 | 32,41 |
| PRO.T01.F22.S4 | D11 | 1 | 1:10 | 32,84 |
| PRO.T01.F23.S4 | B4 | 6 | 2:1 | 31,51 |
| PRO.T01.F23.S4 | B10 | 6 | 1:10 | 32,59 |
| PRO.T01.F24.S3 | E1 | 3 | 2:1 | 40 |
| PRO.T01.F24.S3 | E5 | 3 | 1:1 | 40 |
| PRO.T01.F24.S3 | E9 | 3 | 1:10 | 30,65 |
| PRO.T01.F25.S4 | E2 | 5 | 2:1 | 40 |
| PRO.T01.F25.S4 | E6 | 5 | 1:1 | 34,26 |
| PRO.T01.F25.S4 | E10 | 5 | 1:10 | 42,74 |
| PRO.T01.F27.S4 | C4 | 6 | 2:1 | 32,42 |
| PRO.T01.F27.S4 | C10 | 6 | 1:10 | 32,4 |
| PRO.T01.F29.S3 | A1 | 6 | 2:1 | 33,06 |
| PRO.T01.F29.S3 | A7 | 6 | 1:1 | 34,78 |
| PRO.T01.F30.S3 | F2 | 6 | 2:1 | 31,44 |
| PRO.T01.F30.S3 | F8 | 6 | 1:1 | 33,32 |
| PRO.T01.F32.S3 | H3 | 3 | 2:1 | 36,75 |
| PRO.T01.F32.S3 | H7 | 3 | 1:1 | 31,8 |
| PRO.T01.F32.S3 | H11 | 3 | 1:10 | 37,87 |
| PRO.T01.F32.S4 | G2 | 3 | 2:1 | 40 |
| PRO.T01.F32.S4 | G6 | 3 | 1:1 | 35,9 |
| PRO.T01.F32.S4 | G10 | 3 | 1:10 | 34,49 |
| PRO.T01.F33.S4 | B3 | 5 | 2:1 | 37,94 |
| PRO.T01.F33.S4 | B7 | 5 | 1:1 | 40 |
| PRO.T01.F33.S4 | B11 | 5 | 1:10 | 33,46 |
| PRO.T01.F34.S3 | D4 | 1 | 2:1 | 33,75 |
| PRO.T01.F34.S3 | D8 | 1 | 1:1 | 34,73 |
| PRO.T01.F34.S3 | D12 | 1 | 1:10 | 22,56 |
| PRO.T01.F35.S3 | G1 | 6 | 2:1 | 31,93 |
| PRO.T01.F35.S3 | G7 | 6 | 1:1 | 34,82 |
| PRO.T01.F35.S4 | C2 | 1 | 2:1 | 36,35 |
| PRO.T01.F35.S4 | C6 | 1 | 1:1 | 37,46 |
| PRO.T01.F35.S4 | C10 | 1 | 1:10 | 40 |
| PRO.T01.F39.S4 | F1 | 5 | 2:1 | 34,96 |
| PRO.T01.F39.S4 | F5 | 5 | 1:1 | 36,1 |
| PRO.T01.F39.S4 | F9 | 5 | 1:10 | 35,85 |
| qPCR BLANK | G12 | 1 | 1:10 | 40 |
| qPCR BLANK | H12 | 1 | 1:10 | 40 |
| qPCR BLANK | G12 | 5 | 1:10 | 40 |
| qPCR BLANK | H12 | 5 | 1:10 | 40 |
| SYN.Feed 1 | E6 | 6 | 1:1 | 25,75 |
| SYN.Feed 1 | E12 | 6 | 1:10 | 26,92 |
| SYN.Feed 2 | F6 | 6 | 1:1 | 25,67 |
| SYN.Feed 2 | F12 | 6 | 1:10 | 25,51 |
| SYN.Feed 3 | G6 | 6 | 1:1 | 25,4 |
| SYN.Feed 3 | G12 | 6 | 1:10 | 26,3 |
| SYN.T01.F01.S4 | A3 | 5 | 2:1 | 39,83 |
| SYN.T01.F01.S4 | A7 | 5 | 1:1 | 40 |
| SYN.T01.F01.S4 | A11 | 5 | 1:10 | 37,99 |
| SYN.T01.F02.S4 | H1 | 6 | 2:1 | 32,76 |
| SYN.T01.F02.S4 | H7 | 6 | 1:1 | 34,63 |
| SYN.T01.F04.S3 | F1 | 1 | 2:1 | 32,89 |
| SYN.T01.F04.S3 | F5 | 1 | 1:1 | 33,98 |
| SYN.T01.F04.S3 | F9 | 1 | 1:10 | 24,96 |
| SYN.T01.F04.S4 | G2 | 6 | 2:1 | 33,77 |
| SYN.T01.F04.S4 | G8 | 6 | 1:1 | 34,66 |
| SYN.T01.F05.S3 | H3 | 1 | 2:1 | 38,14 |
| SYN.T01.F05.S3 | H7 | 1 | 1:1 | 32,87 |
| SYN.T01.F05.S3 | H11 | 1 | 1:10 | 21,2 |
| SYN.T01.F05.S4 | C1 | 3 | 2:1 | 40 |
| SYN.T01.F05.S4 | C5 | 3 | 1:1 | 29,77 |
| SYN.T01.F05.S4 | C9 | 3 | 1:10 | 34,24 |
| SYN.T01.F06.S4 | H4 | 5 | 2:1 | 36,97 |
| SYN.T01.F06.S4 | H8 | 5 | 1:1 | 36,17 |
| SYN.T01.F10.S4 | D1 | 5 | 2:1 | 28,02 |
| SYN.T01.F10.S4 | D5 | 5 | 1:1 | 33,52 |
| SYN.T01.F10.S4 | D9 | 5 | 1:10 | 36,49 |
| SYN.T01.F11.S4 | D3 | 3 | 2:1 | 34,17 |
| SYN.T01.F11.S4 | D7 | 3 | 1:1 | 34,66 |
| SYN.T01.F11.S4 | D11 | 3 | 1:10 | 34,93 |
| SYN.T01.F12.S4 | E2 | 1 | 2:1 | 40 |
| SYN.T01.F12.S4 | E6 | 1 | 1:1 | 29,87 |
| SYN.T01.F12.S4 | E10 | 1 | 1:10 | 40 |
| SYN.T01.F22.S3 | G1 | 5 | 2:1 | 36,88 |
| SYN.T01.F22.S3 | G5 | 5 | 1:1 | 40 |
| SYN.T01.F22.S3 | G9 | 5 | 1:10 | 37,05 |
| SYN.T01.F23.S3 | A3 | 3 | 2:1 | 40 |
| SYN.T01.F23.S3 | A7 | 3 | 1:1 | 40 |
| SYN.T01.F23.S3 | A11 | 3 | 1:10 | 36,06 |
| SYN.T01.F23.S4 | A2 | 6 | 2:1 | 28,43 |
| SYN.T01.F23.S4 | A8 | 6 | 1:1 | 32,85 |
| SYN.T01.F29.S4 | E4 | 3 | 2:1 | 40 |
| SYN.T01.F29.S4 | E8 | 3 | 1:1 | 40 |
| SYN.T01.F29.S4 | E12 | 3 | 1:10 | 35,93 |
| SYN.T01.F33.S3 | C3 | 1 | 2:1 | 35,16 |
| SYN.T01.F33.S3 | C7 | 1 | 1:1 | 40 |
| SYN.T01.F33.S3 | C11 | 1 | 1:10 | 33,69 |
| SYN.T01.F34.S3 | F1 | 3 | 2:1 | 28,68 |
| SYN.T01.F34.S3 | F5 | 3 | 1:1 | 38,49 |
| SYN.T01.F34.S3 | F9 | 3 | 1:10 | 33,48 |
| SYN.T01.F36.S4 | G3 | 3 | 2:1 | 37,69 |
| SYN.T01.F36.S4 | G7 | 3 | 1:1 | 37,18 |
| SYN.T01.F36.S4 | G11 | 3 | 1:10 | 36,28 |
| SYN.T01.F38.S3 | B1 | 5 | 2:1 | 36,94 |
| SYN.T01.F38.S3 | B5 | 5 | 1:1 | 33,67 |
| SYN.T01.F38.S3 | B9 | 5 | 1:10 | 32,92 |
| Water sample 1 | A5 | 6 | 1:1 | 26,99 |
| Water sample 1 | A11 | 6 | 1:10 | 29,61 |
| Water sample 2 | B5 | 6 | 1:1 | 28,17 |
| Water sample 2 | B11 | 6 | 1:10 | 25,12 |
| Water sample 3 | C5 | 6 | 1:1 | 30,68 |
| Water sample 3 | C11 | 6 | 1:10 | 31,57 |
| Water sample 4 | D5 | 6 | 1:1 | 30,51 |
| Water sample 4 | D11 | 6 | 1:10 | 27,82 |
| Water sample 5 | E5 | 6 | 1:1 | 27,13 |
| Water sample 5 | E11 | 6 | 1:10 | 24,23 |

**Supplementary Table S2.2 Tag combinations for all PCR products used in this study.**

See Excel file “Suppl_Table_S2_2.xlsx”.

## 2.2 Metagenomic data generation

Extraction of DNA for metagenomics were carried out using ZymoBiomics DNA miniPrep (Cat. D4300). Fragmentation of metagenomic DNA were carried out, using Covaris M220 with microTUBE-50 AFA Fiber Screw-Cap. 50µl DNA were fragmented. Samples was normalised to 400 ng input for library preparation. Library preparation was based on Single-tube library preparation for degraded DNA(Carøe et al. 2018) and a modified version for BGI sequencing(Mak et al. 2017). Libraries were built with 32µl DNA input. Preparation is based on three steps, including: end-repair, ligase of adapters, and fill-in. All steps are processed on ice (4°C) in order to minimize chimeric DNA.

Prior the indexing of libraries, all libraries were analysed with quantitative PCR (qPCR) to estimate optimal cycle settings on a Mx3005P qPCR System (Agilent Technologies). qPCR was performed on purified libraries, using 1µl library in a 20x dilution and a Taq mastermix containing 0.1U/µl AmpliTaq Gold™ (Applied Biosystems, USA) with 1x Gold PCR buffer (Applied Biosystems, USA), 2.5 mM MgCl2 (Applied Biosystems, USA), 20mg/ml Bovine Serum Albumin (BSA) (Bio Labs), 1µl SYBR/ROX stock solution, 0.25mM dNTP, 0.2 µM forward BGI index-primer and reverse BGI index-primer(Mak et al. 2017) to a final concentration of 20µl. qPCR was performed on an Agilent Technologies Mx3005 instrument with the following cycling conditions: 95°C for 10 min, followed by 40 cycles of 95°C for 30 seconds, 60°C for 60 seconds, and 72°C for 60 seconds. This was followed by 95°C for 60 seconds, 55°C for 30 seconds and 95°C for 30 seconds to make a dissociation curve.

Purified libraries were indexed and amplified for sequencing, using customized index primers for MGISeq-2000. PCR was performed in triplicates of 80µl reactions using 10µl DNA template, 1× AmpliTaq Gold buffer, 2.5 mM MgCl2, 0.16mg Bovine Serum Albumin (BSA), 0.25 mM dNTP, 0.2mM common forward and reverse indexed primers specific for each sample, and 0.1U AmpliTaq Gold polymerase. Libraries were amplified in an Applied Biosystems 2720 Thermal Cycler using the following conditions: 95°C for 12 min, followed by a number of cycles of 95°C for 20 s, 60°C for 30 s, and 72°C for 40 seconds, followed by 5 min at 72°C. Libraries were given 7 cycles of PCR based on Ct values assessed from the qPCR. Amplification was followed by a purification step, using magnetic SPRI-beads[^20^](https://paperpile.com/c/CRX0QO/y1hu). Indexed libraries were sequenced using 150PE on a MGIseq-2000 at BGI Europe, where one lane of sequencing was used for each library, please find all sample information below.

**Supplementary Table S2.3) Sample information for metagenomic processed samples**

| **Sample Name** | **Feed Code** | **Feed** | **Article Name** | **Gut Section** | **Fulton K Category** | **Procedural Name** | **qPCR Cycles** |
| --- | --- | --- | --- | --- | --- | --- | --- |
| εT01.F23.S4.W17 | Epsilon | Control | Ctrl_M01 | Mid | >2 | 1 | 8 |
| βT01.F20.S3.W17 | Beta | Probiotics | Pro_D01 | Distal | <2 | 12 | 8 |
| ζT01.F33.S3.W17 | Zeta | Synbiotics | Syn_D01 | Distal | <2 | 25 | 8 |
| εT01.F11.S4.W17 | Epsilon | Control | Ctrl_M02 | Mid | <2 | 37 | 8 |
| εT01.F23.S3.W17 | Epsilon | Control | Ctrl_D01 | Distal | >2 | 41 | 8 |
| βT01.F07R.S4.W17 | Beta | Probiotics | Pro_M02 | Mid | >2 | 46R | 8 |
| εT01.F11.S3.W17 | Epsilon | Control | Ctrl_D02 | Distal | <2 | 51 | 8 |
| βT01.F07.S3.W17 | Beta | Probiotics | Pro_D02 | Distal | >2 | 5 | 8 |
| ζT01.F12.S3.W17 | Zeta | Synbiotics | Syn_D02 | Distal | >2 | 75 | 8 |
| βT01.F20R.S4.W17 | Beta | Probiotics | Pro_M01 | Mid | <2 | 85R | 8 |
| ζT01.F12.S4.W17 | Zeta | Synbiotics | Syn_M02 | Mid | >2 | 87 | 8 |
| ζT01.F33.S4.W17 | Zeta | Synbiotics | Syn_M01 | Mid | <2 | 99 | 8 |

**Supplementary Table S2.4) Data availability information of metagenomic processed data**

| **Sample Name** | **Feed** | **Gut Section** | **Fulton K Category** | **Origin** | **ENA Archive** |
| --- | --- | --- | --- | --- | --- |
| Ctrl_M01 | Control | Mid | >2 | Rasmussen et al. 2021a | PRJEB40990 |
| Pro_D01 | Probiotics | Distal | <2 | Rasmussen et al. 2021a | PRJEB40990 |
| Syn_D01 | Synbiotics | Distal | <2 | This Study | PRJEBXXXX |
| Ctrl_M02 | Control | Mid | <2 | Rasmussen et al. 2021a | PRJEB40990 |
| Ctrl_D01 | Control | Distal | >2 | Rasmussen et al. 2021a | PRJEB40990 |
| Pro_M02 | Probiotics | Mid | >2 | This Study | PRJEBXXXX |
| Ctrl_D02 | Control | Distal | <2 | Rasmussen et al. 2021a | PRJEB40990 |
| Pro_D02 | Probiotics | Distal | >2 | This Study | PRJEBXXXX |
| Syn_D02 | Synbiotics | Distal | >2 | This Study | PRJEBXXXX |
| Pro_M01 | Probiotics | Mid | <2 | This Study | PRJEBXXXX |
| Syn_M02 | Synbiotics | Mid | >2 | This Study | PRJEBXXXX |
| Syn_M01 | Synbiotics | Mid | <2 | This Study | PRJEBXXXX |

## 2.3 Metabolomic extraction and preparation

A subset of ten samples from each of the three feeding types was selected according to Fulton's condition factor (five random samples below K=2, and five samples above K=2), resulting in a total sample size of 30 samples. In order to minimise batch effects all samples were randomised prior to any laboratory processing.

Samples were homogenised in 100 % methanol (MeOH) in a 1:10 sample:solvent ratio. Homogenisation was carried in an OMNI Bead Ruptor 24, using dry ice in order to keep homogenised samples around 0°C to minimise degradation of metabolites during homogenisation.

Six procedural blanks were included in homogenisation. A volume of 100 µl of all samples were collected into Quality Control samples (QC samples), due to Quality assessment after ultrahigh pressure liquid chromatography–mass spectrometry (UHPLC-MS/MS).

In order to enhance detection of metabolites, all samples were purified after homogenisation, using solid phase extraction (SPE). The SPE was carried out conditioning with 100 % MeOH and washing with 0.1 % Formic Acid. Samples were eluted in 100 % MeOH. Samples were concentrated using SpeedVac and resuspended in 5% MeOH. In order to correct for biases related to injection order, samples were divided into two replicates and ordered in an anti-parallel order prior to UHPLC-MS/MS.

Metabolites were detected and quantified on a Q Exactive™ HF Hybrid Quadrupole-Orbitrap™ Mass Spectrometer. An amount of 21.4 µL of sample was loaded on a preconcentration trap (a C18 100 µm × 5 mm cartridge) and eluted onto an analytical column (75 µm × 250 mm, 2 µm C18) with a chromatographic triple-phasic 53 min gradient ranging from 1% to 64% mobile phase B (98% acetonitrile and 0.1% formic acid) at 300 nL per minute. The total analysis time was 65 min, and mobile phase A consisted of 2% acetonitrile and 0.1% formic acid. The high-resolution mass spectrometer was operated with positive electrospray ionisation in data-dependent mode by automatically switching between MS and MS/MS fragmentation. Based on a survey MS scan in the Orbitrap, operated at a mass resolution of 120,000 at m/z 200 with a target of 3e6 ions and a maximum injection time at 50 ms, the twelve most intense peptide ions were selected for MS/MS fragmentation in subsequent scans. The selected ions were isolated (in a m/z 1.4 window) and higher-energy collision dissociation was done at a normalized collision energy, and fragments recorded in centroid mode at a resolution of 60,000 (m/z 200) with a 250 ms max filling time and target of 1e5 ions.

# 3 Analytical procedures

## 3.1 Feed Performance of rainbow trout

Feed performance parameters were analysed for each group based on recorded bulk fish weight, numbers of individuals and consumed feed during the experimental feeding period for each of the five replicate tanks in each experimental feed group.

The following parameters were calculated, as previously described (Villumsen et al. 2020) and as below:

1. Gain of weight (%):

$$GW =((Final weight (g) - Initial weight (g))/Initial weight (g)) x 100\%$$

2. Feed conversion ratio and Feed Efficiency Ratio:

$$FCR =Consumed feed (g)/(Final weight (g) - Initial weight (g))$$

$$FER=2-FCR$$

3. Protein efficiency ratio:

$$PER = Weight gain (g)/Consumed protein (g)$$

4. Lipid efficiency ratio:

$$LER = Weight gain (g)/Consumed fat (g)$$

5. Specific growth rate:

$$SGR =((ln(Final weight (g)) - ln(Initial weight (g))/Duration of feeding (days)) x 100$$

6. Specific feed rate:

$$SFR = FCR x SGR$$

**Supplementary Figure S1: Performance Test of feeding trial.** A) Radar plot of Lipid efficiency Ratio, Specific Feed Conversion Ratio, Specific Growth Rate, Feed Conversion Ratio, Gain of Weight (%), and Protein Efficiency Ratio between feeding types (CTRL, PRO, and SYN). The degree by which Protein Efficiency Ratio and Lipid Efficiency Ratio respectively explain the key growth parameter Gain of weight and Feed Conversion Ratio are shown as correlation analysis (B-E). Feeding groups were visualised as orange for control feed, blue for probiotic additive, and red for synbiotic additive.

## 3.2 Bacterial 16S rRNA gene profiling of Taxonomy and compositional analysis

Please see: <https://github.com/JacobAgerbo/Multi_Omic_Rainbow_Trout/tree/main/16S> for details of 16S analysis.

**Supplementary Figure S2. Overview of bacterial 16S metabarcoding data processing. A)** Boxplots of proportion of retained reads throughout steps of the DADA2 pipeline. **B)** Rarefaction curves of species (ASVs) and sequencing depth.

## 3.3 Metagenomic bioinformatics and analysis

Please see: <https://github.com/JacobAgerbo/Multi_Omic_Rainbow_Trout/tree/main/Metagenomics> for details of metagenomic analysis.

**Supplementary Figure S3. Collector’s curves of all paired-end reads for metagenome of rainbow trout.** Saturation of curves indicate saturated read depth for 5X coverage of novel reads in percentages. Colours indicate feeding types, as indicated in legend.

**Supplementary Table S3.1) Q2-Q3 Coverage and read recruitment of metagenomic reads of bin (Lactobacillus) and MAGs (*Mycoplasma salmoninae mykiss* and Enterobacteriaceae)**

|  | **Q2-Q3 Coverage** | | | **Read Recuitment (%)** | | |
| --- | --- | --- | --- | --- | --- | --- |
| **Sample** | **Lactobacillus** | **Mycoplasma salmoninae mykiss** | **Unknown Enterobacteriaceae** | **Lactobacillus** | **Mycoplasma salmoninae mykiss** | **Unknown Enterobacteriaceae** |
| CTRL_D01 | 0.5 | 847.3 | 0.0 | 27.7 | 68.7 | 3.6 |
| CTRL_D02 | 0.2 | 317.8 | 0.0 | 36.9 | 56.4 | 6.8 |
| CTRL_M01 | 6.4 | 5939.9 | 0.0 | 14.1 | 84.5 | 1.4 |
| CTRL_M02 | 8.4 | 3667.5 | 2.4 | 20.2 | 76.8 | 3.0 |
| PRO_D01 | 0.0 | 0.0 | 4.8 | 61.0 | 16.9 | 22.1 |
| PRO_D02 | 0.0 | 0.0 | 0.0 | 72.4 | 17.4 | 10.2 |
| PRO_M01 | 0.0 | 0.0 | 0.0 | 66.3 | 20.5 | 13.2 |
| PRO_M02 | 0.0 | 0.0 | 4.1 | 66.8 | 20.8 | 12.4 |
| SYN_D01 | 0.2 | 589.1 | 0.0 | 33.7 | 60.9 | 5.4 |
| SYN_D02 | 0.0 | 0.1 | 0.0 | 70.6 | 13.9 | 15.5 |
| SYN_M01 | 0.3 | 0.2 | 5.2 | 67.0 | 18.8 | 14.2 |
| SYN_M02 | 0.0 | 0.0 | 0.0 | 70.0 | 15.2 | 14.9 |

Further information of the metagenomic bin and MAGs can be found in a online available summary on figshare: <https://figshare.com/s/cdee30247b82787f953c> and will be publicly available upon acceptance at: 10.6084/m9.figshare.13193846.

### 3.3.1 Enrichment Analysis

Enrichment analysis for metagenome between CTRL, PRO, and SYN was carried out using anivo. Test was based on GLMs, using *r-package DESeq2* in R. Please find code and details, at: <https://github.com/JacobAgerbo/Multi_Omic_Rainbow_Trout/upload/main/Metagenomics/Differential_Test>.

**Supplementary Table S3.2 Output of differential enrichment analysis, using GLMs with *DESeq2*.**

See Excel file “Suppl_Table_S3_2.xlsx”.

## 3.4 Metabolomic analysis and metabolite network substructural analysis

***Please see:*** [*https://github.com/JacobAgerbo/Multi_Omic_Rainbow_Trout/tree/main/Metabolomics*](https://github.com/JacobAgerbo/Multi_Omic_Rainbow_Trout/tree/main/Metabolomics) *for details of metabolomic analysis and refer to main text about details.*

### 3.4.1 GNPS Quality Control and workflow of UHPLC-MS/MS data

Emperor plots for raw data composition revealing extraction and procedural blanks clustering away from samples, indicating different compositions between blanks and samples. Furthermore, we see Quality Control pools (QCs) are clustering in the middle between samples on second and third axis, indicating QCs are rather unaffected by sample variance, which we hypothesise as a result of a successful pooling of sampling into QCs, where most metabolites from samples were found in QCs.


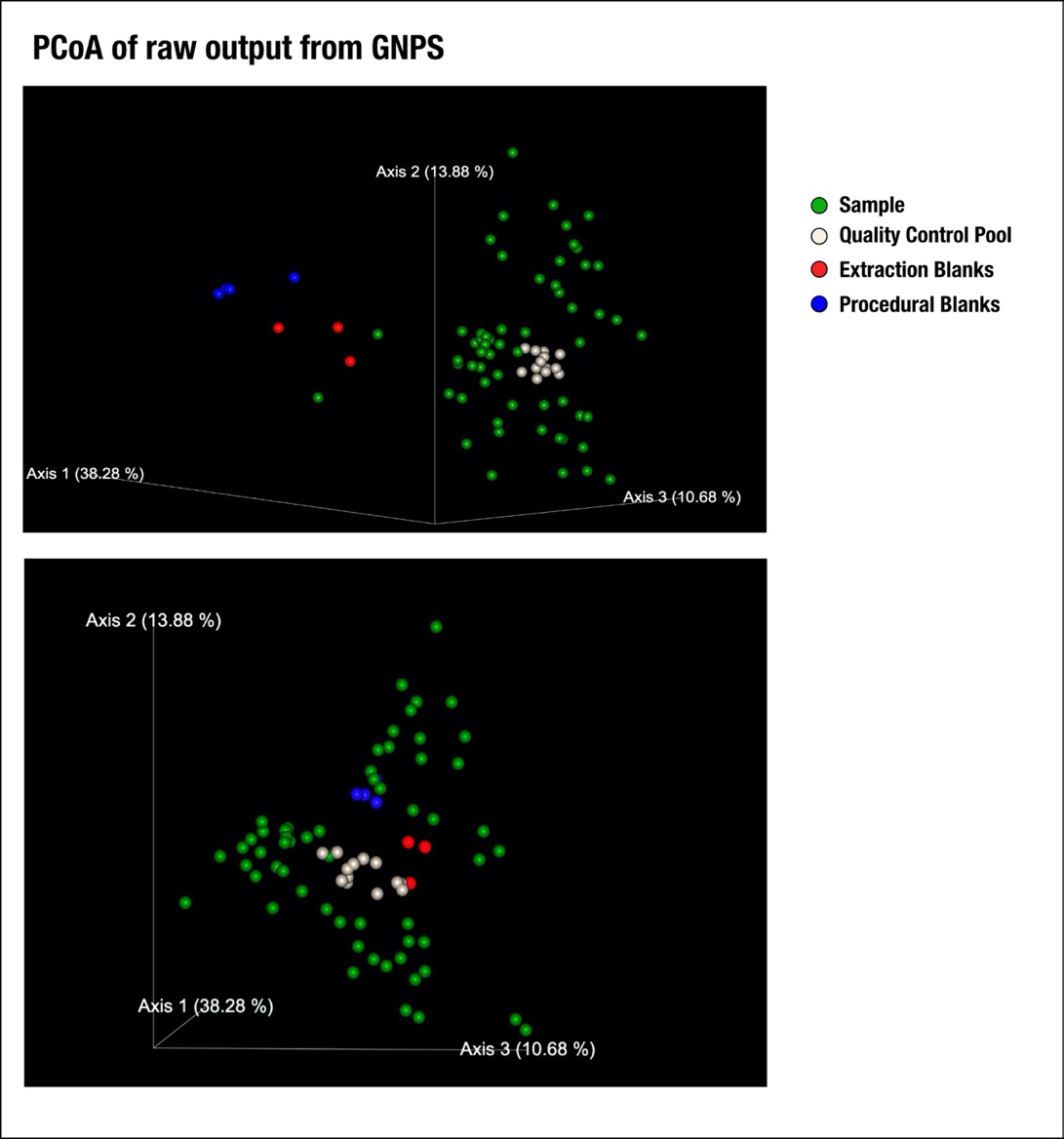


**Supplementary Figure S4. Initial raw composition of metabolites after GNPS processing**. Emperor plot of metabolites across sample types, including samples, quality pools, extraction blanks, and procedural blanks. Colours are indicated in legend. Link for interactive qiime2 emperor plot can be found at: <https://gnps.ucsd.edu/ProteoSAFe/status.jsp?task=afb39ec5e61c4c6b9414cdce65b1993b>.

ThermoFisher Scientific UHPLC-Orbitrap-MS/MS RAW files were converted into mzML files using Proteo Wizard (Adusumilli and Mallick 2017). A molecular network was created using the classical molecular networking workflow <https://ccms-ucsd.github.io/GNPSDocumentation> on the Global Natural Product Social Molecular Networking (GNPS) platform http://gnps.ucsd.edu (Wang et al. 2016; Rogers et al. 2019; Watrous et al. 2012). The data were filtered by removing all MS/MS fragment ions within +/- 17 Da of the precursor m/z. MS/MS spectra were window filtered by choosing only the top 6 fragment ions in the +/- 50 Da window throughout the spectrum. The precursor ion mass tolerance was set to 1.0 Da and a MS/MS fragment ion tolerance of 0.02 Da. A network was then created where edges were filtered to have a cosine score above 0.7 and more than 4 matched peaks. Further, edges between two nodes were kept in the network only if each of the nodes appeared in each other's respective top 10 most similar nodes. Finally, the maximum size of a molecular family was set to 100 nodes, and the lowest scoring edges were removed from molecular families until the molecular family size was below this threshold. The spectra in the network were then searched against all GNPS' spectral libraries. The library spectra were filtered in the same manner as the input data. All matches kept between network spectra and library spectra were required to have a score above 0.7 and at least 4 matched peaks.

In order to enhance identification of unknown metabolites, unsupervised substructures were discovered using MS2LDA(Rogers et al. 2019; van der Hooft et al. 2016), and MS2 spectra were annotated in silico using Network Annotation Propagation (NAP) (da Silva et al. 2018). Furthermore, peptidic natural products (PNPs) were annotated in silico, using DEREPLICATOR (Mohimani et al. 2017). Chemical classes were retrieved for all GNPS library hits and in silico structures using ClassyFire (Djoumbou Feunang et al. 2016). Finally, all structural annotations were combined within one network using MolNetEnhancer (Ernst et al. 2019). Further annotation of metabolites was carried out, using MetDNA (Shen et al. 2019). Networks were visualised using Cytoscape/v3.8.0 83.

Annotation of BAMs were carried out with a combination of GNPS network and SIRIUS4 with CSI:FingerID (Dührkop et al. 2019, 2015).

**GNPS Network Analysis jobs, are publicly available at:**

*GNPS Network:* <https://gnps.ucsd.edu/ProteoSAFe/status.jsp?task=afb39ec5e61c4c6b9414cdce65b1993b>

*MS2LDA:* <https://gnps.ucsd.edu/ProteoSAFe/status.jsp?task=6abde09ef6ea434fa2c26b2303a9c7c4>

*Dereplicator:*

<http://gnps.ucsd.edu/ProteoSAFe/status.jsp?task=a3740a3245a44943867101fce3280fe7>

*NAP - M+NA:* <https://proteomics2.ucsd.edu/ProteoSAFe/status.jsp?task=6f0e74ca9b0147fbb61801762b6f8545>

*NAP - M+H:* <https://proteomics2.ucsd.edu/ProteoSAFe/status.jsp?task=1d55e6c6fe0f4905a9c154a78e135e17>

### 3.4.2 Differential intensity analysis of unknown metabolites of UHPLC-MS/MS data

Differential analysis of unknown metabolites from UHPLC-MS/MS was carried out using *R package MetaboDiff.* Prior to analysis were metabolites filtered according to presence in blanks and only metabolites present in 50 of 60 samples were kept, resulting in 741 metabolites. Metabolites were normalised with variance stabilising normalisation (VSN) and imputed with k-nearest neighbour (knn) with a cut-off on 0.4.

**Supplementary Figure S5. Differential intensity analysis of unknown metabolites.** Volcano plot of a differential intensity test across feeding types, which were based on 741 metabolites with a spectral match to known compounds. Metabolites with an adjusted p-value below 0.05 were considered to be significant. Colour of nodes are dependent on adjusted p-value, where red nodes were significantly different between feeding types (p.adj < 0.05) and gray nodes are insignificant (p.adj > 0.05).

Distribution of significant metabolites were analysed across feeding types (CTRL, PRO, and SYN).

Heatmaps of unknown metabolites were constructed from most significant abundant metabolites derived from differential metabolomic analysis, using MetaboDiff (detailed in Supplementary information 3.3). Hierarchical clustering of metabolites and characterisation of PAMs were carried out, using the R-package cluster.

**Supplementary Figure S6: Alterations of metabolomic landscape across feeding types and Mycoplasma relative abundance.** Heatmap of 168 metabolites, which were significantly different among feeding types. The dendrogram shows clustering of samples based on similarity of their overall metabolite profiles. Coloured boxes below the dendrogram show for each sample I) feeding types, where CTRL is visualised as orange, PRO as red, and SYN as blue, II) relative abundance of Mycoplasma in each sample. Below is shown plots indicating density of SDs across metabolites for each sample. The heatmap shows the variance stabilising normalised (VSN) intensity. Colours indicate standard deviations (SDs) from mean VSN intensity and show whether a given metabolite is under (blue) or over (red) represented in an individual sample relative to the mean across all samples. Samples and metabolites were clustered based on partitioning around medoids (PAMs) as indicated in the legend. Putative metabolite classes are shown for in silico annotated metabolites. Each column in the heatmap indicates fish individuals from each feeding type.

### 3.4.3 Compound discoverer workflow of IC HR-MS/MS data

We performed retention time alignment, unknown compound detection, and compound grouping across all samples. Furthermore, we conducted prediction of elemental compositions for all compounds, fills gaps across all samples, and hides chemical background (using Blank samples). Identification of compounds using mzCloud (ddMS2) and ChemSpider (formula or exact mass), and performed similarity search for all compounds with ddMS2 data using mzCloud. We applied mzLogic algorithm to rank order ChemSpider results. We applied QC-based batch normalization based on 13 quality control (QC) samples, using Compound Discoverer 3.2.0.421 (Thermo Scientific). Please see Supplementary Table S3.3 for full workflow of Compound Discoverer.

**Supplementary Table S3.3 Compound Discoverer workflow for IC HR-MS/MS untargeted metabolomics**

See Excel file “Suppl_Table_S3_3.xlsx”.

### 3.4.4 Biological inferring of IC HR-MS/MS and UHPLC-MS/MS data

Precursor mass from annotated compounds from UHPLC-MS/MS and IC HR-MS/MS data, using full match on GNPS and mzcloud respectively were imputed, using *R package Metabodiff* and were subsequently quantile normalised.

For mzcloud annotated compounds only compounds with a “full match” to mzcloud database were included and all compounds from GNPS were included from UHPLC-MS/MS. Duplicates and isomers of known compounds were summed to minimise redundancy, resulting in 309 annotated compounds. To do biological inferring of annotated compounds from UHPLC-MS/MS and UHPIC-MS/MS data we used *BioCyc* (Karp et al. 2019) and *MetaCyc* (Caspi et al. 2020) databases to display enrichment of compounds across pathways.

***Please see:***

<https://biocyc.org/dashboard/dashboard.html?st=biocyc13-50326-3823338459>

In order to test differential intensity across feeding types, we conducted a pairwise Wilcoxon test with FDR correction between feeding types for each of the 309 known compounds.

**Supplementary Table S3.4 Summary of differential intensity analysis of known compounds from UHPLC-MS/MS and IC HR-MS/MS**

See Excel file “Suppl_Table_S3_4.xlsx”.

**Supplementary Table S3.5 Output of differential abundance test of UHPLC-MS/MS, using *Metadiff* based on the 741 VSN metabolites**

See Excel file “Suppl_Table_S3_5.xlsx”.

### 3.4.5 Association between bacterial ASVs abundances and metabolites

#### Model fitting

After quantifying the ASVs and obtaining the metabolite profiles in our samples, we computed the correlation between the relative abundance of the ASVs per sample to the relative abundances of a subset of the metabolites. First, we restricted our analysis to the 26 samples that had their microbiome profiled using the 16S amplicon sequencing and had metabolites measured using MS/MS. Of these 26 samples, including 10 from CTRL, 9 from PRO, and the remaining 7 from the SYN group.

We filtered out the rare ASVs, and retained only the six most abundant ASVs to restrict our analyses to only the most relevant ASVs, where we would have statistical power to detect associations. To reduce the effect of excess missing data for metabolites, we used zero inflation in the metabolite observations, by excluding the metabolites with more than five zero observations out of the 26 samples, resulting in a final set of 569 metabolites. The zero observations were coded as missing values. Finally, we quantile normalized the metabolite relative abundances to a standard normal to ensure that the assumptions underlying the linear models used for the association between ASV abundances and metabolite relative abundances were met.

To measure the putative effect of ASVs on metabolite relative abundances, for each metabolite, we used a stepwise regression procedure, using *R-package MASS*, where we started with a linear model where the abundances of all six ASVs were used as explanatory variables. At each step of the stepwise regression, the ASV that explained the minimum proportion of the variance of the metabolite relative abundances was removed, followed by checking all the ASVs currently excluded from the model for significant improvement in model fit. Akaike information criteria (AIC) was used to decide the removal and re-inclusion of the ASVs in the linear model. The final model was selected when no ASVs could be removed without significant reduction in explanatory power of the model, and no ASVs could be added with significant improvement of fit.

Finally, note that we did not include feed type as an explanatory variable for the metabolite relative abundances, since the feed type was highly correlated with the ASV abundance information. Specifically, the abundance of *Mycoplasma* was indicative of control vs. non-control feed type. Thus, given the modest sample sizes in this study, we decide to focus our test on the putative effects of ASV abundance on metabolite relative abundances.

#### Model testing and multiple testing correction

A total of 569 metabolites were modelled using the ASV abundances. For each metabolite, we tested the final model obtained from the stepwise procedure using a F-statistic to test the proportion of variance explained by the chosen ASVs. Since we conducted 569 such F-statistic based tests to test the goodness of fit of the linear model, we used bonferroni correction at a significance threshold of 0.05 (corrected p-value 8.79 x 10^-5^) to detect metabolites that where examined further for their relationships with observed abundance of ASVs.

**Supplementary Table S3.6 Output of F statistics from association test between relative abundance of ASVs and intensity of metabolites**

See Excel file “Suppl_Table_S3_6.xlsx”.

**Supplementary Table S3.7 Node table for 350 metabolites used to infer the top 25 most bacterial associated metabolites**

See Excel file “Suppl_Table_S3_7.xlsx”.

# References

Adusumilli, Ravali, and Parag Mallick. 2017. “Data Conversion with ProteoWizard MsConvert.” *Methods in Molecular Biology*  1550: 339–68.

Alberdi, Antton, Ostaizka Aizpurua, |. M. Thomas, P. Gilbert, and Kristine Bohmann. 2018. “Scrutinizing Key Steps for Reliable Metabarcoding of Environmental Samples.” *Methods in Ecology and Evolution / British Ecological Society* 9: 134–47.

Carøe, Christian, Shyam Gopalakrishnan, Lasse Vinner, Sarah S. T. Mak, Mikkel Holger S. Sinding, José A. Samaniego, Nathan Wales, Thomas Sicheritz-Pontén, and M. Thomas P. Gilbert. 2018. “Single-Tube Library Preparation for Degraded DNA.” Edited by Susan Johnston. *Methods in Ecology and Evolution / British Ecological Society* 9 (2): 410–19.

Caspi, Ron, Richard Billington, Ingrid M. Keseler, Anamika Kothari, Markus Krummenacker, Peter E. Midford, Wai Kit Ong, Suzanne Paley, Pallavi Subhraveti, and Peter D. Karp. 2020. “The MetaCyc Database of Metabolic Pathways and Enzymes - a 2019 Update.” *Nucleic Acids Research* 48 (D1): D445–53.

Djoumbou Feunang, Yannick, Roman Eisner, Craig Knox, Leonid Chepelev, Janna Hastings, Gareth Owen, Eoin Fahy, et al. 2016. “ClassyFire: Automated Chemical Classification with a Comprehensive, Computable Taxonomy.” *Journal of Cheminformatics* 8 (November): 61.

Dührkop, Kai, Markus Fleischauer, Marcus Ludwig, Alexander A. Aksenov, Alexey V. Melnik, Marvin Meusel, Pieter C. Dorrestein, Juho Rousu, and Sebastian Böcker. 2019. “SIRIUS 4: A Rapid Tool for Turning Tandem Mass Spectra into Metabolite Structure Information.” *Nature Methods* 16 (4): 299–302.

Dührkop, Kai, Huibin Shen, Marvin Meusel, Juho Rousu, and Sebastian Böcker. 2015. “Searching Molecular Structure Databases with Tandem Mass Spectra Using CSI:FingerID.” *Proceedings of the National Academy of Sciences of the United States of America* 112 (41): 12580–85.

Ernst, Madeleine, Kyo Bin Kang, Andrés Mauricio Caraballo-Rodríguez, Louis-Felix Nothias, Joe Wandy, Christopher Chen, Mingxun Wang, et al. 2019. “MolNetEnhancer: Enhanced Molecular Networks by Integrating Metabolome Mining and Annotation Tools.” *Metabolites* 9 (7). https://doi.org/10.3390/metabo9070144.

Hooft, Justin Johan Jozias van der, Joe Wandy, Michael P. Barrett, Karl E. V. Burgess, and Simon Rogers. 2016. “Topic Modeling for Untargeted Substructure Exploration in Metabolomics.” *Proceedings of the National Academy of Sciences of the United States of America* 113 (48): 13738–43.

Karp, Peter D., Richard Billington, Ron Caspi, Carol A. Fulcher, Mario Latendresse, Anamika Kothari, Ingrid M. Keseler, et al. 2019. “The BioCyc Collection of Microbial Genomes and Metabolic Pathways.” *Briefings in Bioinformatics* 20 (4): 1085–93.

Mak, Sarah Siu Tze, Shyam Gopalakrishnan, Christian Carøe, Chunyu Geng, Shanlin Liu, Mikkel Holger S. Sinding, Lukas F. K. Kuderna, et al. 2017. “Comparative Performance of the BGISEQ-500 vs Illumina HiSeq2500 Sequencing Platforms for Palaeogenomic Sequencing.” *GigaScience* 6 (8): 1–13.

Mohimani, Hosein, Alexey Gurevich, Alla Mikheenko, Neha Garg, Louis-Felix Nothias, Akihiro Ninomiya, Kentaro Takada, Pieter C. Dorrestein, and Pavel A. Pevzner. 2017. “Dereplication of Peptidic Natural Products through Database Search of Mass Spectra.” *Nature Chemical Biology* 13 (1): 30–37.

Rogers, Simon, Cher Wei Ong, Joe Wandy, Madeleine Ernst, Lars Ridder, and Justin J. J. van der Hooft. 2019. “Deciphering Complex Metabolite Mixtures by Unsupervised and Supervised Substructure Discovery and Semi-Automated Annotation from MS/MS Spectra.” *Faraday Discussions* 218 (0): 284–302.

Shen, Xiaotao, Ruohong Wang, Xin Xiong, Yandong Yin, Yuping Cai, Zaijun Ma, Nan Liu, and Zheng-Jiang Zhu. 2019. “Metabolic Reaction Network-Based Recursive Metabolite Annotation for Untargeted Metabolomics.” *Nature Communications* 10 (1): 1516.

Silva, Ricardo R. da, Mingxun Wang, Louis-Félix Nothias, Justin J. J. van der Hooft, Andrés Mauricio Caraballo-Rodríguez, Evan Fox, Marcy J. Balunas, Jonathan L. Klassen, Norberto Peporine Lopes, and Pieter C. Dorrestein. 2018. “Propagating Annotations of Molecular Networks Using in Silico Fragmentation.” *PLoS Computational Biology* 14 (4): e1006089.

Villumsen, Kasper Rømer, Maki Ohtani, Torunn Forberg, Elisabeth Aasum, John Tinsley, and Anders Miki Bojesen. 2020. “Synbiotic Feed Supplementation Significantly Improves Lipid Utilization and Shows Discrete Effects on Disease Resistance in Rainbow Trout (Oncorhynchus Mykiss).” *Scientific Reports* 10 (1): 16993.

Wang, Mingxun, Jeremy J. Carver, Vanessa V. Phelan, Laura M. Sanchez, Neha Garg, Yao Peng, Don Duy Nguyen, et al. 2016. “Sharing and Community Curation of Mass Spectrometry Data with Global Natural Products Social Molecular Networking.” *Nature Biotechnology* 34 (8): 828–37.

Watrous, Jeramie, Patrick Roach, Theodore Alexandrov, Brandi S. Heath, Jane Y. Yang, Roland D. Kersten, Menno van der Voort, et al. 2012. “Mass Spectral Molecular Networking of Living Microbial Colonies.” *Proceedings of the National Academy of Sciences of the United States of America* 109 (26): E1743-52.
